# A mind-brain-body dataset of MRI, EEG, cognition, emotion, and peripheral physiology in young and old adults

**DOI:** 10.1038/sdata.2018.308

**Published:** 2019-02-12

**Authors:** Anahit Babayan, Miray Erbey, Deniz Kumral, Janis D. Reinelt, Andrea M. F. Reiter, Josefin Röbbig, H. Lina Schaare, Marie Uhlig, Alfred Anwander, Pierre-Louis Bazin, Annette Horstmann, Leonie Lampe, Vadim V. Nikulin, Hadas Okon-Singer, Sven Preusser, André Pampel, Christiane S. Rohr, Julia Sacher, Angelika Thöne-Otto, Sabrina Trapp, Till Nierhaus, Denise Altmann, Katrin Arelin, Maria Blöchl, Edith Bongartz, Patric Breig, Elena Cesnaite, Sufang Chen, Roberto Cozatl, Saskia Czerwonatis, Gabriele Dambrauskaite, Maria Dreyer, Jessica Enders, Melina Engelhardt, Marie Michele Fischer, Norman Forschack, Johannes Golchert, Laura Golz, C. Alexandrina Guran, Susanna Hedrich, Nicole Hentschel, Daria I. Hoffmann, Julia M. Huntenburg, Rebecca Jost, Anna Kosatschek, Stella Kunzendorf, Hannah Lammers, Mark E. Lauckner, Keyvan Mahjoory, Ahmad S. Kanaan, Natacha Mendes, Ramona Menger, Enzo Morino, Karina Näthe, Jennifer Neubauer, Handan Noyan, Sabine Oligschläger, Patricia Panczyszyn-Trzewik, Dorothee Poehlchen, Nadine Putzke, Sabrina Roski, Marie-Catherine Schaller, Anja Schieferbein, Benito Schlaak, Robert Schmidt, Krzysztof J. Gorgolewski, Hanna Maria Schmidt, Anne Schrimpf, Sylvia Stasch, Maria Voss, Annett Wiedemann, Daniel S. Margulies, Michael Gaebler, Arno Villringer

**Affiliations:** 1 Department of Neurology, Max Planck Institute for Human Cognitive and Brain Sciences, Leipzig, Germany; 2 MindBrainBody Institute at the Berlin School of Mind and Brain, Humboldt-Universität Berlin, Berlin, Germany; 3 International Max Planck School on the Life Course, Max Planck Institute for Human Development, Berlin, Germany; 4 International Max Planck Research School NeuroCom, Max Planck Institute for Human Cognitive and Brain Sciences, Leipzig, Germany; 5 Max Planck Research Group for Cognitive and Affective Control of Behavioral Adaptation, Max Planck Institute for Human Cognitive and Brain Sciences, Leipzig, Germany; 6 Lifespan Developmental Neuroscience, Technische Universität Dresden, Dresden, Germany; 7 Department of Neuropsychology, Max Planck Institute for Human Cognitive and Brain Sciences, Leipzig, Germany; 8 Netherlands Institute for Neuroscience, Amsterdam, Netherlands; 9 Spinoza Centre for Neuroimaging, Amsterdam, Netherlands; 10 IFB Adiposity Diseases, Leipzig University Medical Center, Leipzig, Germany; 11 Department of Psychology, University of Haifa, Haifa, Israel; 12 Nuclear Magnetic Resonance Group, Max Planck Institute for Human Cognitive and Brain Sciences, Leipzig, Germany; 13 Day Clinic for Cognitive Neurology, University Hospital Leipzig, Leipzig, Germany; 14 Department of Psychology, University Leipzig, Leipzig, Germany; 15 Database Management, Max Planck Institute for Human Cognitive and Brain Sciences, Leipzig, Germany; 16 Max Planck Research Group for Neuroanatomy & Connectivity, Max Planck Institute for Human Cognitive and Brain Sciences, Leipzig, Germany; 17 Library, Max Planck Institute for Human Cognitive and Brain Sciences, Leipzig, Germany; 18 Institute for Laboratory Medicine, Clinical Chemistry and Molecular Diagnostics (ILM) of the Medical Faculty at the Leipzig University, Leipzig, Germany; 19 Department of Psychology, Stanford University, Stanford, California 94305, USA; 20 Leipzig Research Centre for Civilization Diseases LIFE, Leipzig University, Leipzig, Germany

**Keywords:** Human behaviour, Emotion, Functional magnetic resonance imaging, Electroencephalography - EEG

## Abstract

We present a publicly available dataset of 227 healthy participants comprising a young (N=153, 25.1±3.1 years, range 20–35 years, 45 female) and an elderly group (N=74, 67.6±4.7 years, range 59–77 years, 37 female) acquired cross-sectionally in Leipzig, Germany, between 2013 and 2015 to study mind-body-emotion interactions. During a two-day assessment, participants completed MRI at 3 Tesla (resting-state fMRI, quantitative T1 (MP2RAGE), T2-weighted, FLAIR, SWI/QSM, DWI) and a 62-channel EEG experiment at rest. During task-free resting-state fMRI, cardiovascular measures (blood pressure, heart rate, pulse, respiration) were continuously acquired. Anthropometrics, blood samples, and urine drug tests were obtained. Psychiatric symptoms were identified with Standardized Clinical Interview for DSM IV (SCID-I), Hamilton Depression Scale, and Borderline Symptoms List. Psychological assessment comprised 6 cognitive tests as well as 21 questionnaires related to emotional behavior, personality traits and tendencies, eating behavior, and addictive behavior. We provide information on study design, methods, and details of the data. This dataset is part of the larger MPI Leipzig Mind-Brain-Body database.

## Background & Summary

Emotions - intrinsically related to the body - have a huge influence on our behavior^1^. The connection between emotions and the body has been acknowledged by “folk psychology”^2^, language metaphors (e.g., “heart-breaking”), and scientists: in classic theories, emotions arise from our perception of bodily changes, which is understood as more^3^ or less strongly influenced^4^ by cognitive-evaluative processes. Hence, emotions – like other mental processes – depend on interactions between the brain and the rest of the body. While in psychology, a lot of research measured the physiological effects of psychological manipulations, the inverse (body-mind) direction has been less frequently studied^5^. In clinical research, the opposite is true: While psychological changes after physical or somatic illness, such as depressive symptoms after stroke^6^ or a cancer diagnosis^7–9^ have been investigated, mental factors contributing to bodily diseases have received comparatively less scientific recognition. For example, psychological stress has negative influences on somatic and mental health^10^ and emotional episodes like depressive symptoms have been discussed as a risk factor for stroke^11^, coronary heart disease^12^ or diabetes^13^.

Informed by these recent studies, we investigate psychological factors that play a key role in the pathogenesis, development, and treatment of somatic diseases in a multi-modular approach. This “mind-body-emotion” approach emphasizes the bi-directionality of brain-body interactions as they underlie mental phenomena and the importance of psychological factors for somatic health and disease. In the “Leipzig Study for Mind-Body-Emotion Interactions” (LEMON), we acquired a large dataset of physiological, psychological, and neuroimaging measures in younger and older healthy adults.

The LEMON dataset provides the following advantages:

1. Subjects underwent an extensive medical and psychological selection procedure: Past and current somatic or mental illnesses as well as current medication status are well-controlled and documented. Careful adherence to selection and “health” criteria is especially important when investigating healthy aging.
2. Psychometric tools to assess cognitive and socio-emotional characteristics are tailored to relate them to bodily and brain measures. The LEMON dataset thereby enables basic research on the healthy interaction between brain, mind, and body – as it is assumed to be altered in somatic and mental illness.
3. LEMON complements data of brain structure and brain function with extensive bodily measures. Measures of peripheral physiology serve a double function of being utilized for removing artifacts from the Magnetic Resonance Imaging (MRI) data (as e.g., the fMRI BOLD signal is also influenced by magnetic field changes induced by peripheral fluctuations^14^). However, in addition to explaining psychological variance for themselves, they can be related to cerebral measures to test for fundamental brain-body interactions at rest (e.g., heart rate variability and fMRI data^15^).
4. The current study included a broad set of psychological measures to cover individual psychological categories but also their overlap. This is important as psychological categories are sometimes artificially separated, which hinders their comprehensive investigation. This holds true for broader fields like cognition and emotion^16^ but also for more specific psychological processes like emotion regulation and value-based decision-making^17^. Particularly from a clinical viewpoint, a more integrative approach is beneficial, as risk factors for disease usually do not occur in isolation – and for example mental distress, hypertension, and obesity often co-occur^18–20^.

In summary, the LEMON dataset is particularly suited to comprehensively relate cognitive and emotional traits or states to physiological characteristics of brain and body. While focusing on fundamental mind-body-emotion interactions in healthy younger and older adults, our data and results may inform clinical research. Here, we present the study’s objectives, design, and methods together with available data types, their quality and quantities.

The dataset presented here was acquired as one of two complementary data acquisition protocols on a partially overlapping cohort of participants which constitute the MPI-Leipzig Mind-Brain-Body database. All MRI data of both projects were acquired on the same scanner. Taken in conjunction with the data acquired in the complementary project by Mendes *et al*.^21^, the MPI-Leipzig Mind-Brain-Body (MPILMBB) database aims to enable exploration of individual variance across a wide range of cognitive, emotional, physiological phenotypes in relation to the brain.

## Methods

### Participants

The total sample included 227 participants in two age groups. The young age group was between 20–35 years old (N=153, 45 females, median age=24 years, mean age=25.1 years, standard deviation (SD)=3.1) and the older age group was between 59–77 years old (N=74, 37 females, median age=67 years, mean age=67.6 years, SD=4.7). All participants were tested at the Day Clinic for Cognitive Neurology of the University Clinic Leipzig and the Max Planck Institute for Human and Cognitive and Brain Sciences (MPI CBS) in Leipzig, Germany. The study was carried out in accordance with the Declaration of Helsinki and the study protocol was approved by the ethics committee at the medical faculty of the University of Leipzig (reference number 154/13-ff).

### Recruitment and Exclusion Criteria

**Figure 1:**
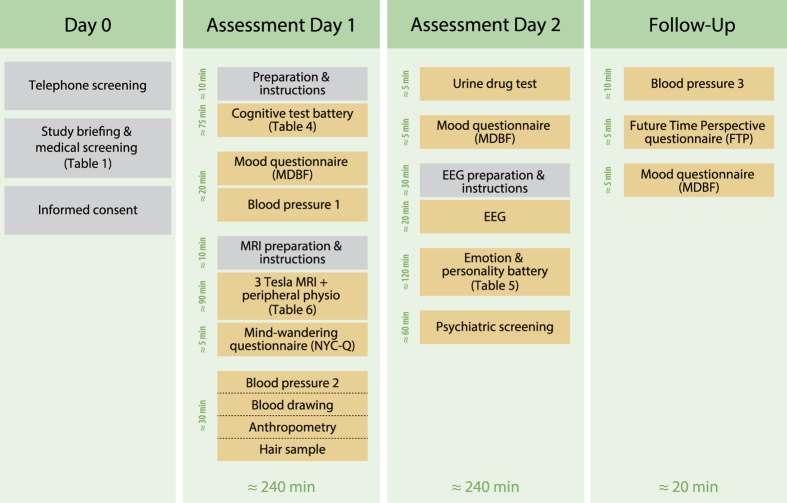
Overview of data acquisition. Measures are listed in their order of acquisition and time duration on each assessment day.

Participants were recruited via public advertisements, leaflets, online advertisements, and information events at the University of Leipzig. Eligibility for the study was determined in two steps that are referred to as Day 0 in Fig. 1. First, we prescreened prospective participants via telephone with a semi-structured interview for study eligibility (N=695). Individuals that did not meet any exclusion criteria in the prescreening (Table 1) were invited to MPI CBS to receive detailed information about the study in a group briefing. There, they were informed about the study procedure and its measures with a special focus on MRI acquisition and safety. Following the group briefing, the study physician performed a second, individual screening of every participant to ensure that none of the exclusion criteria were fulfilled. Participants who were included in the study provided written informed consent prior to any data acquisition for the study (including agreement to their data being shared anonymously). Participants received monetary compensation for volunteering in the study after the completion of all assessment days. A participant was excluded because of brain pathology after completion of study, thus the total number of included participants became 227.

### Procedure

Data acquisition was performed from September 2013 until September 2015 and distributed over four “rounds” (subsamples) with varying time intervals between each round. Round 1 was acquired from 09/2013-12/2013 and included 53 (34 females, young N=31, 17 females, mean age=24.0, SD=2.8, older N=22, 17 females, mean age=67.4, SD=4.1, 23.3% of total sample) participants.

Acquisition of round 2 lasted from 02/2014-06/2014 and included 59 (25 females, young N=36, 14 females, mean age=25.3, SD=3.3, older N=23, 11 females, mean age=68.9, SD=5.2, 26% of total sample) participants.

In round 3, 58 (23 females, young N=29, 14 females, mean age=25, SD=3.7, older N=29, 9 females, mean age=66.6, SD=4.6, 25.6% of total sample) participants were tested between 10/2014 and 03/2015.

Round 4 consisted of 57 young males only (mean age=25.6, SD=2.6, 25.1% of total sample) and was acquired from 03/2015-09/2015. In round 4 we limited the sample to only male participants, since these participants were included in a follow-up stress experiment (not described here) which only included males (this was due to the attempt to replicate a previous study performed in male soldiers).

The following general study procedure was established. During the process of the study some measures were adapted and are thus not available for the total sample. Table 2 and Table 3 give a detailed overview of all measures and their availability for assessment day 1 and day 2 and Table 4 gives detailed overview of all measures and their availability for follow-up assessment days.

Participants completed two assessment days of approximately 4 hours duration each (Fig. 1). The first assessment day (day 1) included a cognitive test battery, MRI scanning, blood pressure and anthropometric measurements as well as acquisition of a blood sample. On the second assessment day (day 2), we acquired resting-state electroencephalogram (EEG) data and participants completed a psychological assessment including an emotion and personality test battery as well as a psychiatric interview. Participants were also invited for follow-up experiments, of which some measures are included here (3rd occasion of blood pressure, Future Time Perspective questionnaire, Multidimensional Mood State Questionnaire).

A complementary project by Mendes *et al*.^21^ included 194 participants of which 109 participants completed both protocols which enables repeated-measures (e.g., test-retest) analyses. Some data from Mendes *et al*. will be released as part of the study described here (e.g. continuous peripheral physiological recordings during resting-state (rs) fMRI).

### Psychological Assessment

#### Cognitive Test Battery

Cognitive tests were administered by undergraduate psychology students specifically trained in neuropsychological assessment following a standardized protocol. On day 1, participants underwent cognitive testing session of six cognitive tests in a fixed order (cf. Table 5). The subtests (“Alertness”, “Incompatibility”, and “Working Memory”) of the “Test of Attentional Performance” (TAP) were administered electronically via computer. An overview of this cognitive testing session is shown in Table 5, and detailed information on all measures is provided in the subsequent section.

##### California Verbal Learning Task (CVLT)

The California Verbal Learning Task (CVLT)^22^ assesses verbal learning and memory capacity. Participants are acoustically presented with 16 words, which have to be memorized and recalled or recognized several times. By quantifying, the CVLT provides how much information has been acquired over the rounds and — by generating a variety of measures — it can provide information about different learning strategies. The task has two main parts (CVLT-part 1 and CVLT-part 2) and for the second part another free recall takes place after 20 min. During the interval between recall 1 and 2, typically a different non-verbal task is administered, for instance other cognitive tasks. In the present study the TAP-Test (see below) was administered, because the items are not supposed to interfere with verbal learning.

##### Test of Attentional Performance (TAP)

Test of Attentional Performance (TAP)^23^ measures different aspects of attentional processing. Here, the TAP version 2.3.1 was used. Three subtests assessed a participant’s capacity of sustained attention (“TAP Alertness”, and “TAP Incompatibility”- i.e. Simon effect) and working memory (“TAP Working Memory”- i.e. 2-back task)^23^. Mistakes, omissions, and reaction times in these subtests were recorded as measures of performance.

##### Trail Making Test (TMT)

The Trail Making Test (TMT) measures cognitive flexibility, and it consists of subtest A (TMT-A) and subtest B (TMT-B)^24^. Participants are asked to quickly and correctly connect circles which are randomly distributed on a piece of paper. In TMT-A, these circles contain numbers from 1 to 25. In TMT-B, numbers and letters have to be connected in alternating and increasing order. The reaction time quantifies visual attention and executive functioning.

##### Wortschatztest (WST)

The Vocabulary Test (Wortschatztest, WST)^25^ indicates the measurement of verbal intelligence level and the assessment of language comprehension. By determining the vocabulary of a person, the WST allows estimation of his/her crystallized intelligence. It consists of 43 rows with 6 words each. In each row, participants have to identify the one word that actually exists in German.

##### Subtest 3 of the “Leistungsprüfsystem 2” (LPS-2)

Subtest 3 of the Performance Testing System (Leistungsprüfsystem 2, LPS-2)^26^ measures logical or inferential thinking and quantifies fluid intelligence. In subtest 3, participants are asked to identify the one item in a series of symbols that does not follow the logical rule of that series. The goal is to find as many items as possible within three minutes.

##### Regensburger Wortflüssigkeitstest (RWT)

The Regensburger Word Fluency Test (Regensburger Wortflüssigkeitstest, RWT)^27^ quantifies the verbal fluency of a person. In the section of “S-Words”, participants have two minutes to name as many valid German words as possible that start with the letter “S”. In the “Animals” section, as many animals as possible should be named within two minutes. The correct number of words quantifies formal lexical (“S-Words”) or categorical-semantic fluency (“Animals”).

### Emotion and Personality Test Battery

On day 2, participants were asked to answer electronic version of 18 emotion-related questionnaires (*cf. first18 sections below)* in a randomized sequence on a computer (LimeSurvey version 2.0)^28^. The whole questionnaire completion took on average 1.5 hours to 2.5 hours with a short break after 45 min.

Besides those electronic testing sesion of 18 questionnaires, which were answered on a computer via LimeSurvey, there were pen-and-paper version of three other emotion-related questionnaires (*cf. last 3 sections below)* that were filled out at different time points. The Multidimensional Mood State Questionnaire (*German* MDBF) was answered on each of the two assessment days. After the MRI scanning session, participants filled out the New York Cognition Questionnaire (NYC-Q). The questionnaire of Future Time Perspective (FTP) was assessed during LEMON Rounds 1–3 only at the beginning of a follow-up experiment. An overview of the individual questionnaires can be found in Table 6 (available online only) and a more detailed description is given in the section below.

#### Big-Five of Personality (NEO-FFI)

We used the German adaptation of NEO-Five-Factor Inventory^29^ to assess Costa and McCrae’s Big-Five of Personality Inventory (NEO-FFI)^30^. The 60 items can be divided into the five factors of “Neuroticism”, “Extraversion”, “Openness to experience”, “Agreeableness”, and “Conscientiousness”. Answers are given on a 5-point Likert scale ranging from 0 (strong denial) to 4 (strong approval).

#### Impulsive Behavior Scale (UPPS)

We applied the German adaptation (UPPS)^31^ of Impulsive Behavior Scale (UPPS)^32^ to assess the four sub-dimensions of impulsivity “Urgency”, “Premeditation”, “Perseverance” and “Sensation Seeking”. The 45 items are rated on a 4-point Likert scale ranging from 1 (agree strongly) to 4 (disagree strongly).

#### Behavioral Inhibition and Approach System (BIS/BAS)

The German version^33^ of the Behavioral Inhibition and Approach System (BIS/BAS)^34^ was applied to measure reactivity of the aversive “Behavioral Inhibition” and the appetitive “Behavioral Approach” motivational systems in response to punishment or reward. This measure consists of a BIS subscale and three BAS subscales “Drive”, “Reward Responsiveness”, and “Fun Seeking”, each consisting of 7 items. A total of 24 items are rated on a 4-point Likert-type response format ranging from 1 (does not apply to me at all) to 4 (fully applies to me).

#### Emotion Regulation Questionnaire (ERQ)

To measure inter-individual differences in habitual emotion regulation, participants completed the German version^35^ of the emotion regulation questionnaire (ERQ)^36^, which has 10 items that are answered on a 7-point Likert-type scale ranging from 1 (strongly disagree) to 7 (strongly agree). Six of the 10 items measure the tendency to use reappraisal for emotion regulation, and the other 4 items assess habitual expressive suppression.

#### Cognitive Emotion Regulation Questionnaire (CERQ)

The Cognitive Emotion Regulation Questionnaire (CERQ) evaluates the cognitive aspects of emotion regulation^37,38^. It contains nine scales that measure five adaptive (acceptance, positive refocusing, refocusing on planning, positive reappraisal, putting into perspective) and four maladaptive emotion regulation strategies (self blame, rumination, catastrophising, blaming others) on a 5-point Likert scale from 0 (almost never) to 4 (almost always).

#### Affect Regulation Style (MARS)

We used the German version (external official translation, not validated yet) of the Measure of Affect Regulation Style (MARS)^39^ to evaluate cognitive and behavioral aspects of emotion regulation. The scale consists of six subscales of “Behavioral Distraction”, “Cognitive Distraction”, “Situation-focused Strategies”, “Affect-focused Strategies”, “Disengagement”, and “Avoidance”. Ratings are given on a 7-point Likert scale ranging from 0 (not at all) to 6 (almost always). Since this data refers to the first version of a German validation — a process which is still on-going — the data should be used with caution.

#### Social Support Questionnaire (F-SozU K-22)

Perceived social support was assessed using the German *Fragebogen zur Sozialen Unterstützung*^40^, the 22-item short version Social Support Questionnaire (F-SozU K-22)^41^. The scale comprises subscales of “Emotional Support”, “Practical support”, “Social Integration”, “Availability of Trusted Person”, and “Satisfaction with Social Support”. The 22 items are answered on a 5-point Likert scale ranging from 1 (does not apply at all) to 5 (strongly applies).

#### Multidimensional Scale of Perceived Social Support (MSPSS)

The German version of the Multidimensional Scale of Perceived Social Support (MSPSS)^42^ was used to evaluate the perceived availability of social resources in the area of friends, family and significant others. In addition to the three subscales of the sources of social support, a sum score can be computed. Ratings can be provided on a 7-point Likert scale ranging from 1 (not true at all) to 7 (very true).

#### Coping Orientations to Problems Experienced (Brief COPE)

We used the German adaptation of the 28-item version of Brief COPE Inventory^43^ to assess participants’ Coping Orientations to Problems Experienced (Brief COPE)^44^. The measure consists of four subscales of “Positive Coping”, “Active Coping”, “Support Coping”, and “Evasive Coping”. The answers are rated on a 4-point Likert scale ranging from 1 (not at all) to 4 (very much).

#### Optimism Pessimism Questionnaire-Revised (LOT-R)

The German version^45^ of Life Orientation Test-Revised (LOT-R) was used to assess individual differences in generalized optimism versus pessimism^46^. The 10 items are added to an overall optimism score ranging from 0–24, with higher scores representing greater positive expectation. Answers are rated on a 5-point Likert scale ranging from 0 (does not apply at all) to 4 (strongly applies).

#### Perceived Stress Questionnaire (PSQ)

We used the German version^47^ of 20-item short version of the Perceived Stress Questionnaire (PSQ)^48^ in order to assess the perception, appraisal, and processing of stressors during the last two years. The scale contains four subscales of “Worries”, “Tension”, “Joy”, and “Demands”. Answers are rated on a 4-point Likert scale from 1 (almost never) to 4 (usually).

#### Trier Inventory of Chronic Stress (TICS)

To assess aspects of chronic stress we applied the German version^49^ of the Trier Inventory of Chronic Stress (TICS)^50^. The 57-item scale comprises nine factors of chronic stress: “Work Overload”, “Social Overload”, “Pressure to Perform”, “Work Discontent”, “Excessive Demands at Work”, “Lack of Social Recognition”, “Social Tension”, “Social Isolation”, and “Chronic Worrying”. Answers are rated on a 5-point Likert scale ranging from 0 (never) to 4 (very often).

#### Eating Behavior (FEV)

The three-factor eating questionnaire (TFEQ)^51^, German version Fragebogen zum Essverhalten (FEV)^52^, was used to assess three domains of eating behavior. ‘Cognitive Restraint of Eating’ measures whether eating behavior is under cognitive, rather than physiological control, ‘Disinhibition of Eating’ measures the lack of control over eating, especially in the presence of tempting external cues or situations, and ‘Susceptibility to Hunger’ measures the experience of prominent and disturbing subjective hunger feelings. The 60 items are answered in different response formats ranging from dichotomous scales (applies, does not apply) to 4-point Likert scales from 1 (always) to 4 (never) or 1 (very much) to 4 (not). Item 58-60 are rated by selecting from a list of behavior descriptions.

#### Addicted Eating Behavior (YFAS)

We applied the German version^53^ of Yale Food Addiction Scale (YFAS)^54^ in order to classify food-dependent eating behavior. Twenty of the total 27 items measure the seven DSM-IV-TR criteria of dependence^55^, two items measure if the eating behavior causes a clinically significant impairment, three items ask for particular foods related to the problematic eating behavior, and three items act as a primer for the other questions. The items are rated either on a 5-point Likert scale from 0 (never) to 4 (four times a week to daily) or dichotomous 0 (never) or 1 (yes).

#### Emotional Intelligence Questionnaire (TEIQue-SF)

The 30-item short version of the Trait Emotional Intelligence Questionnaire (TEIQue-SF)^56^ of German adaptation^57^ was used to measure emotion-related dispositions and self-perception abilities. The scale contains the four subscales of “Well-being“, “Self-control”, “Emotionality”, and “Sociability”, which can be averaged to one “Global Trait Emotional Intelligence” score. Answers are rated in a 7-point Likert format, ranging from 1 (do not agree at all) to 7 (agree completely).

#### State-Trait Anxiety Inventory (STAI-G-X2)

We applied the German version^58^ of the Trait Scale of the State-Trait Anxiety Inventory (STAI-G-X2) short version^59^ for the assessment of a situation-independent general condition of anxiety. This subscale consists of 20 items rated on a 4-point Likert scale ranging from 1 (almost never) to 4 (nearly always).

#### State-Trait Anger Expression Inventory (STAXI)

We used the 44-item German version^60^ of the State-Trait Anger Expression Inventory (STAXI)^61^ to measure the habitual experience, expression, and control of anger. We applied the four trait scales “Trait-anger”, the individual anger-disposition, “Anger-in”, the tendency to suppress and non-verbalization of angry feelings, “Anger-out”, the verbal or physical expression of anger towards others or self, and “Anger-control”, which measures the attempt to control anger-expressions. All ratings were ranked rated on a 4-point Likert scale either from 1 (not at all or hardly ever) to 4 (very much or nearly always).

#### Toronto-Alexithymia Scale (TAS)

The German version^62^ of the 26-item Toronto-Alexithymia Scale (TAS)^63^ was used to measure alexithymia, difficulty experiencing, and expressing emotional states. We applied all three subscales: “difficulty with identifying feelings”, “difficulty with expressing and describing feelings”, and “externally-oriented thinking”. Answers are rated on 5-point Likert scale from 1 (does not apply at all) to 5 (applies completely).

#### Multidimensional Mood State Questionnaire (MDBF)

The 24-item German version of the Multidimensional Mood State Questionnaire (German MDBF)^64^ was completed by the participants on each assessment day. Mood ratings (“happy”, “nervous”, etc.) are ranked on a 5-point Likert scale from 1 (not at all) to 5 (very much). Three subscales can be computed along the dimensions of “good-bad”, “awake-tired”, “calm-nervous”.

#### Future Time Perspective Questionnaire (FTP)

We applied the Future Time Perspective Questionnaire^65^ to assess the individual anticipation of time left to live. Agreement with the statements is ranked on a 7-point Likert scale ranging from 1 (very untrue) to 7 (very true). The mean value indicates the anticipated time horizon.

#### New York Cognition Questionnaire (NYC-Q)

After completion of the scanning session, participants filled out the New York Cognition Questionnaire (NYC-Q)^66^, which measures content and form of self-generated thoughts with 31 statements. The first part “Content of thoughts” is ranked on a Likert scale from 1 (did not describe my thoughts at all) to 9 (completely described my thoughts), while the second part “form of self-generated thoughts” is rated on a scale ranging from 1 (does not characterize my experience at all) to 9 (completely characterizes my experience).

### Assessment of Past and Present Psychiatric Symptoms

Standardized Clinical Interview for DSM IV (SCID-I): The LEMON protocol included a broad characterization of present and past psychiatric symptoms in all participants, which was assessed on the second testing day. Participants underwent SCID^67^ — the Standardized Clinical Interview for Diagnostic and Statistical Manual of Mental Disorders (DSM IV) — to identify whether participants (in the past or in the present) met/meet diagnostic criteria of an Axis 1 psychiatric disorder according to DSM IV^68^. The SCID I is a semi-structured interview that covers the major DSM-IV Axis I diagnoses. Interviews were either led by a trained psychologist or by a psychology student who had been trained to use the SCID I and supervised by a licensed psychiatrist. Documentation includes full current or history of Axis I diagnosis as well as a column with notes on noteworthy current or past subclinical symptoms beyond full fulfillment of diagnostic criteria (e.g., occasional use of an illegal drug, subclinical symptoms).

Screening of Depressive Symptoms or Borderline Symptomatology (HAM-D and BSL-23): Any reported depressive symptoms were additionally assessed by a trained psychologist or trained research assistant using the Hamilton Depression Scale (HAM-D)^69^. Documentation includes the Hamilton sum score. Note that our psychiatric assessment focused primarily on present or past Axis I disorders. In addition, the Borderline Symptoms List (short version BSL-23)^70^ was applied in 170 participants. This questionnaire is a self-rating instrument for borderline-typical symptomatology. Documentation includes sum scores of the BSL-23 and an additional sum score regarding borderline-typical behaviors. Additionally, participants were asked about their relationship status (“yes”/“no”).

Screening for Alcohol Abuse: We also assessed alcohol consumption during the last 28 days using the Time Line Follow Back Questionnaire^71^. Using a calendar, participants self-report retrospectively the number of alcohol units consumed on each day in this period. Documentation includes the number of alcohol units consumed. The Alcohol Use Disorder Identification Test (AUDIT)^72^ questionnaire was administered to screen for any indication of alcohol abuse. We additionally asked for family history of addiction in participants’ 1st to 3rd degree relatives. Documentation includes presence or absence of family history of addiction.

Screening for Substance Abuse: In addition to the semi-structured interview, an in-vitro urine drug screening was performed using the “Multi 8/2 Drogen-Tauchtest” (Diagnostik Nord, Schwerin, Germany) to assess present substance use. The test detects the following substances simultaneously and up to two weeks after their administration: buprenorphine (cut-off 10 ng/mL), amphetamine (cut-off 1000 ng/mL), benzodiazepine (cut-off 300 ng/mL), cocaine (cut-off 300 ng/mL), methamphetamine (cut-off 1000 ng/mL), morphine/heroine (cut-off 300 ng/mL), methadone (cut-off 300 ng/mL), and THC (Marihuana, cut-off 50 ng/mL). Cut-off values of the tests were chosen according to recommendations of the American National Institute on Drug Abuse (NIDA)^73^. Documentation includes name of the substance detected (if any). The drug screening was performed on the second day of assessments, which was randomly assigned in order for participants not to know the date of the urine drug screening ahead of time. Moreover, it covered more than 1 week presence of any substance in urine, thus covering also assessment day 1.

### Physiological data

#### MRI

Magnetic resonance imaging (MRI) was performed on a 3 Tesla scanner (MAGNETOM Verio, Siemens Healthcare GmbH, Erlangen, Germany) equipped with a 32-channel head coil. Over the course of MRI data acquisition, the scanner remained stable and did not undergo any major maintenance or updates which would systematically affect the quality of data provided here. This is also true in relation to the complementary protocol by Mendes *et al*., ensuring comparability between the studies.

##### The imaging protocol lasted approximately 70 min and included the following scans in fixed order

1) gradient echo fieldmap scan for distortion correction in rs-fMRI^74,75^, 2) a pair of spin echo images with reversed phase encoding direction for distortion correction in rs-fMRI^76,77^, 3) rs-fMRI scan, 4) a second pair of spin echo images with reversed phase encoding direction, 5) quantitative and weighted T1 Magnetization-Prepared 2 Rapid Acquisition Gradient Echoes (MP2RAGE)^78^ image, 6) T2-weighted image, 7) Fluid-attenuated inversion recovery (FLAIR) scan, 8) diffusion-weighted imaging (DWI) scan, 9) a pair of spin echo images with reversed phase encoding for distortion correction in DWI, 10) T2*/susceptibility-weighted imaging (SWI) scan.

The data were acquired with a very large coverage using simultaneous multi-slice acquisition to include the brain and the cerebellum. Diffusion data were acquired parallel to the AC-PC line and the volume (149.6 mm height) covered the entire brain including the cerebellum in all participants. The fMRI data were angulated by -15° (backwards) with respect to the AC-PC line. The slice block (147 mm) also covered the entire brain including the full cerebellum. The figures in Supplementary Figure S1 show cross-subject coverage of the fMRI (left) and DWI (right) data normalized to the MNI brain.

During rs-fMRI, electrocardiography (ECG), pulse, beat-to-beat blood pressure and respiration were recorded simultaneously (see section *Continuous peripheral physiological recordings during rs-fMRI*). Before imaging started, participants filled out the first MDBF questionnaire. Once imaging was completed, participants were asked to fill out the New York Cognition Questionnaire (NYC-Q, for details on the questionnaires see section *Emotion and Personality Test Battery*).

##### Resting-state fMRI (rs-fMRI)

A T2^∗^-weighted gradient echo echo planar imaging (EPI) multiband BOLD rs-fMRI scan^79–81^ was acquired to enable functional connectivity analyses. Participants were instructed to remain awake and lie still with their eyes open while looking at a low-contrast fixation cross. Data regarding sleep/wake for the rs-fMRI as such does not exist, but it is assumed that the participants were awake throughout the duration of the scan because they were requested to do so. The sequence parameters were specified as follows: TR=1400 ms and the total number of volumes=657 (for more details see Table 7). The total acquisition time for rs-fMRI was 15 min 30 s. To enable correction for geometric distortions in EPI images from rs-fMRI, a gradient echo fieldmap scan and two pairs of spin echo EPI images with reversed phase encoding direction were acquired.

##### Resting-state fMRI Data Preprocessing

The preprocessing of the rs-fMRI data was implemented in Nipype and the details of it can be found in the complementary project by Mendes *et al*.^21^. The pipeline is available at https://github.com/NeuroanatomyAndConnectivity/pipelines/tree/master/src/lsd_lemon and comprised the following steps: (i) discarding the first five EPI volumes to allow for signal equilibration and steady state, (ii) 3D motion correction (FSL MCFLIRT)^82^, (iii) distortion correction (FSL FUGUE)^83^, (iv) rigid-body coregistration of unwarped temporal mean image to the individual’s anatomical image (FreeSurfer bbregister)^84^, (v) denoising (Nipype rapidart and aCompCor)^85^, (vi) band-pass filtering between 0.01-0.1 Hz (FSL), mean-centering, as well as variance normalization of the denoised time series (Nitime)^86^, (vii) spatial normalization to MNI152 2 mm standard space via transformation parameters derived during structural preprocessing (ANTs SyN)^87^.

### Structural MRI

#### T1 and T2

The MP2RAGE^78^ sequence was acquired for assessment of brain structure with a voxel resolution of 1 mm (isotropic). Resulting T1-weighted images and quantitative T1 maps can be used for analyses of gray and white matter (e.g., cortical thickness, voxel-based morphometry), as well as for the assessment of myelin content^88,89^. Importantly, these T1-weighted images differ from MPRAGE T1-weighted images as they are uniform and free of other imaging properties (i.e. proton density, T2^∗^) which can affect morphometric measurements^90^. The total acquisition time for MP2RAGE was 8 min 22 s. In addition, a standard T2-weighted volume with 1 mm isotropic resolution was acquired within 4 min 43 s (for details see Table 7).

##### T1 Data Preprocessing

The preprocessing of the T1 MP2RAGE data was implemented in Nipype and the details of it can be found in the complementary project by Mendes *et al*.^21^. The pipeline is available at https://github.com/NeuroanatomyAndConnectivity/pipelines/tree/master/src/lsd_lemon and comprised the following steps: The background of the uniform T1-weighted image was removed using CBS Tools^91^, and the masked image was used for cortical surface reconstruction using FreeSurfer’s full version of recon-all^92,93^. A brain mask was created based on the FreeSurfer segmentation results. Diffeomorphic nonlinear registration as implemented in ANTs SyN algorithm^87^ was used to compute a spatial transformation between the individual’s T1-weighted image and the MNI152 1mm standard space. To remove identifying information from the structural MRI scans, a mask for defacing was created from the MP2RAGE images using CBS Tools^91^. This mask was subsequently applied to all anatomical scans.

### Fluid-Attenuated Inversion Recovery (FLAIR)

T2-weighted FLAIR images were used for clinical screening of incidental findings. The scan was changed from a low-resolution 2D FLAIR to a 3D SPACE sequence with fluid-attenuated inversion-recovery preparation after the first 112 participants. Acquisition time for the 2D image was 4 min 42 s and 7 min 2 s for the 3D volume (for details see Table 7).

#### Diffusion-Weighted Imaging (DWI)

We acquired axial whole brain high angular resolution diffusion-weighted images to analyze structural connectivity. The images were acquired with 1.7mm isotropic resolution using a multi-band accelerated sequence^79,81,94^ and an in-plane GRAPPA^95^ (60 diffusion directions, b-value=1000 s/mm^2^, 7 b0 images, for details see Table 7). The total DWI scanning time was 9 min 27 s. To correct for geometric distortions, two volumes with reversed phase encoding (AP and PA) were acquired after the DWI sequence, lasting 1 min 59 s each. A new version of the DWI sequence (CMRR) with a faster calibration procedure was introduced after the first 112 participants which reduced the scanning time to 8 min 38 s and the time for the two scans with reversed phase encoding to 1 min 10 s each.

#### Susceptibility-weighted data acquisition

The visualization of magnetic susceptibility tissue differences is most commonly achieved via gradient echo data acquired using a single- or multi-echo spoiled-gradient-recalled-echo (GRE) sequence^96^. The Susceptibility-Weighted Imaging (SWI) technique capitalizes on the contrast inherent in the magnitude and phase images to improve susceptibility contrast by combining both images to enhance contrast between grey-/white-matter and water/fat, in addition to enhancing the contrast of paramagnetic elements exhibiting high densities in the brain (e.g. iron). SWI has a number of applications in the clinical setting including the diagnosis of cerebral vascular pathology and the detection of abnormal accumulation of mineral deposition. On the other hand, Quantitative Susceptibility Mapping (QSM) is a recently established technique that allows the determination of the intrinsic magnetic susceptibility properties of tissues based on signal from the phase image^97,98^. Susceptibility-weighted data were acquired using a three-dimensional (3D) flow-compensated fast low-angle shot (FLASH) sequence (for parameter details see Table 7) in a sub-sample which was introduced only after 112 participants. High-quality phase maps (i.e. excluding coil-combination pole artifacts) were reconstructed from multi-channel complex signals using an automated, data-driven coil combination method (SVD-ESPIRiT)^99,100^. Both magnitude and phase images are provided for SWI and QSM calculation which could be achieved using varied techniques^96,101^. The total time of acquisition was 7 min 50 s.

### Continuous Peripheral Physiological Recordings During rs-fMRI

During the 15 min 30 s acquisition of resting-state fMRI, continuous beat-to-beat blood pressure (NIBP), electrocardiography (ECG), pulse, and respiration were recorded non-invasively with MR-compatible devices. Blood pressure and pulse via photoplethysmography were recorded with a BIOPAC MP150 acquisition system (BIOPAC Systems Inc., Goleta, CA, USA) and the acquisition software AcqKnowledge (Version 4.0, BIOPAC Systems Inc., Goleta, CA, USA). In addition to the MP150 main hardware unit, blood pressure acquisition required the NIBP-MRI module including a CareTaker Bluetooth® transmitter and pulse acquisition required the OXY100C pulse oximeter module with TSD123A finger clip transducer. All data streams were recorded with a sampling frequency of 1000 Hz. A digital input channel recorded triggers from the MR scanner for synchronisation of blood pressure and pulse data with repetition time onsets of rs-fMRI data.

Beat-to-beat blood pressure was detected from the pulse pressure signal at the brachial artery of the left arm with an air-filled pressure-sensitive sensor. The left arm was supported with tape and cushions to ensure optimal signal quality. The pulse pressure signal was transformed into two continuous streams of systolic and diastolic blood pressure through Pulse Decomposition Analysis^102^.

Initial calibration for the continuous blood pressure acquisition was achieved with a seated resting blood pressure measurement using an automatic oscillometric blood pressure monitor (OMRON M500, OMRON Medizintechnik Handelsgesellschaft mbH, Mannheim, Germany). Blood pressure data was recorded with a sampling frequency of 512 Hz and resampled in AcqKnowledge to 1000 Hz.

ECG and respiration were recorded with an MR-compatible BrainAmp ExG MR amplifier (Brain Products GmbH, Gilching, Germany) with PowerPack battery, SyncBox synchronization interface and relevant sensors (see below), as well as the acquisition software BrainVision Recorder (Version 1.20).

ECG was measured with three reusable ring electrodes that were taped on the participant’s back to reduce artifacts caused by breathing movements of the torso in the magnetic field: the ground electrode was taped at the lumbar region superior to the tailbone (coccyx), the reference electrode was taped at the upper part of the back at the level of the seventh cervical vertebra and the recording electrode was placed on the left-hand side of a participant’s back at the level of the tenth rib.

Respiration was measured with an MR-compatible pneumatic-based respiration sensor within a belt that was fastened around the torso of the participants.

After rs-fMRI was acquired, all sensors were removed from the MR chamber and the MRI session continued without peripheral physiological recordings.

The complementary project by Mendes *et al*.^21^ also comprised rs-fMRI scans with continuous peripheral physiological recordings (as described above). The peripheral physiological data of the 194 participants from Mendes *et al*. will be released as part of the study described here. 109 participants completed both protocols which enables repeated-measures (e.g., test-retest) analyses (see Supplementary Table S1).

For all the above mentioned peripheral physiological parameters only raw data is provided. All available data has been included - irrespective of data quality. The data has been cropped and the MRI artifact was removed but peak detection has not been done. Data quality can be eyeballed from the included image file (^∗^.png) for each participant and modality.

#### EEG

Resting-state EEG (rs-EEG) was obtained in 216 participants who completed the second MDBF just before the EEG recording and underwent the Multi 8/2 drug strip test. The whole experiment session took approximately 1.5 hours (including the 16-minute EEG recording). The raw rs-EEG data with preprocessed rs-EEG and localizer files are openly available.

##### Resting-state EEG

A 16-min rs-EEG was recorded with a BrainAmp MR plus amplifier in an electrically shielded and sound-attenuated EEG booth using 62-channel (61 scalp electrodes plus 1 electrode recording the VEOG below the right eye) active ActiCAP electrodes (both Brain Products GmbH, Gilching, Germany) attached according to the international standard 10–20 extended localization system, also known as 10-10 system,^103^ and referenced to FCz. The ground was located at the sternum and skin electrode impedance was kept below 5 KΩ. The amplitude resolution was set to 0.1 μV. EEG was recorded with a bandpass filter between 0.015 Hz and 1 kHz and digitized with a sampling rate of 2500 Hz. The EEG session comprised a total of 16 blocks, each 60 s long, 8 with eyes-closed (EC) and 8 with eyes-open (EO) (EO and EC segments being interleaved), where the recording started with eyes-closed condition. The blocks were introduced using Presentation software (version 16.5, Neurobehavioral Systems Inc., Berkeley, CA, USA). Participants were seated in front of a computer screen and asked to stay awake while fixating eyes on a black cross presented on a white background (during the eyes-open sessions).

##### Digitized EEG channel locations

Starting from the second round (54th participant), a Polhemus PATRIOT Motion Tracking System (Polhemus, Colchester, VT, USA) localizer was used together with the Brainstorm toolbox^104^ to digitize the exact location of each 62 electrode on a participant’s head relative to three fiducial points (plus 1 electrode referenced to FCz).

##### Resting-State EEG Data Preprocessing

Data from 13 participants were excluded due to missing event information, different sampling rate, mismatching header files or insufficient data quality. The raw EEG data from 203 participants used for preprocessing was downsampled from 2500 Hz to 250 Hz, bandpass filtered within 1-45 Hz (8th order, Butterworth filter) and split into EO and EC conditions for the subsequent analyses. Outlier channels were rejected after visual inspection for frequent jumps/shifts in voltage and poor signal quality. Data intervals containing extreme peak-to-peak deflections or large bursts of high frequency activity were identified by visual inspection and removed. Intervals containing traces from eye blinks or eye movements were not removed at this stage. Further data preprocessing was done in EEGLAB^105^ (version 14.1.1b) for MATLAB (Delorme and Makeig, 2004). The dimensionality of the data was reduced using principal component analysis (PCA), by keeping PCs (N≥30) that explain 95% of the total data variance. Next, independent component analysis^106^ (ICA) was performed using the Infomax (runica) algorithm. Components reflecting eye movement, eye blink or heartbeat related artifacts were removed. Retained independent components for EO (mean: 19.7, range: 9–30) and EC (mean: 21.4, range: 14–28) conditions were back-projected to sensor space for further analysis.

#### Additional Measures.

##### Seated Resting Blood Pressure

Blood pressure (BP) was measured using an automatic oscillometric blood pressure monitor (OMRON M500, OMR HEM-7213-D) and a 22–42 cm arm cuff (OMRON HEM-RML30, both OMRON Medizintechnik Handelsgesellschaft mbH, Mannheim, Germany) after a seated resting period of 5 min. The BP measurements took place on three occasions throughout the course of the study: 1) before the MRI session (BP1), 2) after the MRI session (BP2), 3) at the beginning of follow-up experiments (BP3). All BP measurements were recorded at the left arm. Before the MRI session, an additional measurement at the right arm was recorded to rule out pathologic differences between right and left arm measurements. Accompanying pulse measurements at the arm (Pulse1, Pulse2) were saved during BP measurements 1 and 2. As part of the complementary project by Mendes *et al*.^21^, one blood pressure measurement at the left arm was taken from 91 additional participants before a rs-fMRI session that also included continuous peripheral physiological recordings (see section Continuous Peripheral Physiological Recordings During rs-fMRI).

##### Peripheral Blood Sample Collection and Analysis

A blood sample of approximately 70 ml in total was collected on the first assessment day after acquisition of MRI data. If the blood drawing was not possible on this date, it was acquired on the following assessment days and documented as such. The new date is mentioned in the data files. The blood was collected with four different types of sampling tubes: Serum, EDTA, Citrate and RNA. A portion of the sample was directly sent to the Institute for Laboratory Medicine, Clinical Chemistry and Molecular Diagnostics (ILM) of the Medical Faculty at the Leipzig University; the remaining samples were stored for later use. One serum tube (S-Monovette® 7.5 ml, Sarstedt), one EDTA tube (S-Monovette® 2.7 ml K3E, Sarstedt), and one citrate tube (S-Monovette® 3.0 ml 9NC, Sarstedt) were sent for direct analysis to the ILM. The remaining blood samples were divided into 10 microtainers of 2.0 ml size. Together with three EDTA tubes (S-Monovette® 2.7 ml K3E, Sarstedt) and 2 RNA tubes (Tempus^TM^, Applied Biosystems)—containing stabilization solution—the remaining aliquots were stored at −80 °C for later use.

##### Anthropometry

Classical anthropometric measurements were taken according to standardized procedures by trained medical persons. Body weight was measured using an electronic scale (SECA 813, Seca Gmbh & Co KG) with a precision of 0.01 kg. The participants were barefoot, dressed with empty pockets and without outer garments. Body height of barefoot participants was measured using a stadiometer (SECA 216) to the nearest 0.1 cm. During measurement, the body of the participants were erect and centered placing feet together, the heels and the occiput touching the wall. The waist was measured 1 cm above the belly button, and the hip was measured around the widest part of the hip, with all outer garments removed. The waist and the hip were assessed by using an ergonometric circumference measuring tape (SECA 201) to the nearest 0.1 cm.

##### Hair Sample

To obtain the required amount of hair for the sample the hair had to be a minimum 4 cm long. Participants with colored/dyed hair were also included (as suggested by the analyzing lab), dreadlocked hair was an exclusion criterion. The hair sample was taken from the back of – and as close as possible to – the scalp (posterior vertex position). The strands were carefully placed in aluminum foil and the proximal end was marked. The sample was weighed before being sent to the laboratory. Hair sampling followed the procedure described here: http://poolux.psychopool.tu-dresden.de/dat/videos/hmd1.mpg. Hair samples were delivered to Technische Universität Dresden (TU Dresden) laboratory for analysis (lab of Prof. Dr. C. Kirschbaum). However, the results for cortisol and other hormonal measurements contained an unusual high percentage of 0-values (cortisol 13%, progesterone 63%, corticosterone 73%). Therefore hair-derived corticosteroid measurements were deemed unreliable and will not be released.

### Code Availability

All code that was implemented for MRI data acquisition and processing pipelines is available online:

(https://github.com/NeuroanatomyAndConnectivity/pipelines/tree/v2.0/src/lsd_lemon/). Data handling and computation of summary measures were implemented in Python.

## Data Records

### Data Security and Data Anonymization Procedures

To protect health information prevent direct identification, the participant were given special LEMON IDs. All the data, whether pen-and-paper, computer administered, as well as LimeSurvey, were saved only with these LEMON IDs. For public data sharing we anonymized them once more these IDs into BIDS 6-digit format (010000). Thus our participants are given now IDs such as sub-010000.

For internal use, the data was first saved on a MPI-CBS in-house local secured network. Later, the data for all participants was stored on our instance of the eXtensible Neuroimaging Archive Toolkit (XNAT 35) v.1.6.5. at the MPI-CBS. Access to the initial project was restricted (via XNAT’s private project mode) to members of the Leipzig Study for Mind-Body-Emotion Interactions and Neuroanatomy & Connectivity Group at MPI-CBS for initial curation and quality assessment of data. All data comprised in the MPI-Leipzig Mind-Brain-Body database were derived from MPI-CBS so data import into XNAT was done from our local secured network. A specially customized XNAT uploader was used to upload all participants’ data to XNAT.

The native DICOM format was used for MRI data, whilst a standard ASCII (^∗^.csv, ^∗^.txt) format was employed to upload all other experimental data such as surveys, test batteries, and demographical data in XNAT also in local secured network. The anonymization measures applied to the MRI data consisted of removal of DICOM header tags containing information which could lead to the identification of test participants as well as the defacing of all structural (NIFTI) scans.

This applied mainly for internal use. For releasing the data publicly, the MRI data in NIFTI files in JavaScript Object Notation (*.json) with (*.tsv) format is stored. More details regarding publicly released data can be found below in Usage Notes section.

### MRI Data

All MRI datasets are made available in NIFTI format, and all anatomical scans have been defaced. For more details see Mendes *et al*. The dataset is organized in concordance with the Brain Imaging Data Structure (BIDS) format. This facilitates data analysis, for example with BIDS-Apps^107^ (http://bids-apps.neuroimaging.io). BIDS-Apps encapsulate standard MRI analysis tools within an application that understands the BIDS format and allows automatic access to relevant data and metadata. The MRI raw and preprocessed data can be found in GWDG (https://ftp.gwdg.de/pub/misc/MPI-Leipzig_Mind-Brain-Body-LEMON/) as well as in *Functional Connectomes Project International Neuroimaging Data-Sharing Initiative/Child Mind Institute* (Data Citation 1) and OpenNeuro repository (Data Citation 2).

### EEG Data

The raw rs-EEG data folder contains raw resting state EEG data files (Brain Vision files). The marker codings are S200 for eyes open at rest and S210 for eyes closed at rest.

The preprocessed resting state EEG data folder contains preprocessed EEG (see method section for details) saved in the standard EEGLAB^100^ file format (.set and .fdt files). For each participant (N=203) eyes-closed (EC) and eyes-open (EO) conditions are stored separately thus each having 4 files (2 for EC condition and 2 for EO), with the following naming structure: sub-BIDS condition.fdt (.set) and conditions: eyes closed (EC) or eyes open (EO). This preprocessed data has already been used in another EEG study about non-sinusoidal nature of neuronal oscillations^108^.

The digitized EEG channel locations (62) with Polhemus PATRIOT Motion Tracking System are stored in separate folder as MATLAB (.mat) files. The EEG raw and preprocessed data can be found at GWDG (https://ftp.gwdg.de/pub/misc/MPI-Leipzig_Mind-Brain-Body-LEMON/) or *Functional Connectomes Project International Neuroimaging Data-Sharing Initiative/Child Mind Institute* (Data Citation 1).

### Emotion and Cognition Test Batteries, Assessments, and Other Protocols

The data from most questionnaires are reported as summary scores. Whenever summary scores do not provide an adequate measure, we report raw item scores, for instance the New York cognition (NYC-Q).

Questionnaires that do not come with summary scores are released as raw item scores, namely: New York Cognition Questionnaire (NYC-Q).

Cognitive test data for the CVLT, LPS, TA P, TMT, WST, RWT and emotion and personality test battery questionnaires such as BIS/BAS, CERQ, COPE, ERQ, FEV, F-SozU K-22, LOT-R, MARS, MSPSS, NEO, PSQ, STAI, STAXI, TAS, TEIQue-SF, TICS, UPPS, FTP, YFAS, as well as MDBF and NYC-Q are available via subject-specific ^∗^.csv files. Moreover, for each questionnaire and cognitive test, accompanying specifications and information are given in ^∗^.txt file format with item details and Likert scores.

For each participant, the average age across the course of the study was reported which was the same in both supplementary studies of MPI Leipzig Mind-Brain-Body database. For the purpose of anonymity, the mean age was then binned into five year width (5-year bins). Cutoff values for binning were 20.0, 25.0, 30.0 and so forth. A meta file with demographic summary in ^∗^.csv format includes: gender, age (5-year bins), handedness, formal education, drug test results on day 2, smoking status, SKID, HAM-D, BLS-23, AUDIT, and relationship status. Separate subject-specific (.csv) files with information given in Text (.txt) file include the results of blood sample, blood pressure (for 3 occasions), and anthropometry.

In addition to this meta file, we include a data availability (.csv) file which includes all the LEMON data available for each specific data acquisition section with subscales (1=available, 0=not available).

The data can be accessed via GWDG (https://ftp.gwdg.de/pub/misc/MPI-Leipzig_Mind-Brain-Body-LEMON/), and *Functional Connectomes Project International Neuroimaging Data-Sharing Initiative/Child Mind Institute* (Data Citation 1), or directly at NITRC (https://www.nitrc.org/projects/mpilmbb).

## Technical Validation

Before inclusion in the database, we manually double-checked all datasets for missing or corrupt data. Further quality control of the data was applied to the MRI and behavioral measures, as described below.

### MRI Data Quality Assessment

As described in Mendes *et al*.^21^, we assessed the quality of preprocessed resting-state fMRI images using the mriqc package^109^, implemented in Python. Mriqc creates a report for each individual scan based on different parameters like motion, coregistration, and temporal signal-to-noise (tSNR). The details of tSNR and fieldmap correction can be found in the previous article of Mendes *et al*^21^. Resting-state fMRI data from 8 participants were excluded from preprocessing due to errors during data acquisition (ghosting artifact n=2, incomplete scan n=1), anatomical preprocessing (n=4) or functional preprocessing (n=1).

**Figure 2:**
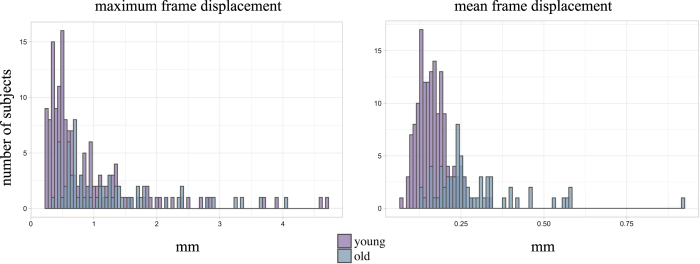
Quality assessment of resting-state fMRI scans. Distribution of motion (maximum and mean framewise displacement).

We visually inspected the quality assessment reports for each participant. Furthermore, frames with high motion were marked according to the framewise displacement, which was calculated as the sum of the absolute values of the six realignment parameters^110^. For comparison, all individual-level scores are displayed with respect to the group-level distribution (N=219). In Fig. 2, the mean and maximum framewise displacement for all participants (N= 219), as well as separately for young (N=152) and old (N=67) participants, are given. Overall, the summary of the motion parameters of our MRI data shows that 91.78% of runs have less than one voxel (2.3 mm) maximum framewise displacement, and mean framewise displacement of 0.202 mm (SD=0.101 mm), demonstrating sufficient quality. Mean framewise displacement was 0.165 mm (SD=0.046 mm) in young participants and a slightly higher in the elderly group (M=0.289, SD=0.134).

### Behavioral Measures Quality Assessment

We calculated descriptive statistics and reliability estimates of each subscale of the emotion and personality battery to ensure their general usability (see Table 6 (available online only)). Since we used mainly German questionnaires with validated factor structures (besides NYC-Q and MARS, which were translated by a professional translator), we report both the Cronbach’s alpha coefficients from the original validation studies of the respective questionnaires as well as the ones calculated from our data in Table 6 (available online only). We did not compute the Cronbach’s alpha coefficient for the NYC-Q as the heterogeneity of items within these questionnaires neither describe a unitary phenomenon nor are they designed to be internal consistent^111^. We recommend a factor analytic approach^66^ to derive behavioral scores from this questionnaire. Moreover, for YFAS we calculated the internal consistency based on Kuder-Richardson’s alpha coefficient^112^.

To further facilitate the evaluation of emotion and personality data, we plotted densities of all subscale scores for younger and older participants (see Supplementary Figure S2), which suggest sensible distributions: For example, normal distributions were observed for most personality traits (NEO-FFI), whereas skewed distributions were observed for social support variables (FSozU, MSPSS). Age-group differences observed after conservative Bonferroni correction for multiple comparisons (α=0.0005) are in general alignment with previous reports of changes in emotional processing during aging^113,114^. Significant differences between younger and older adults emerged on 20 of 98 subscales (see Supplementary Figure S2). These results further underline the value of the LEMON dataset to examine associations of emotions and brain-body functions in healthy aging.

## Usage Notes

The public dataset, protocols, and software used in the acquisition and processing of the data are documented, curated, and available for research purposes. The datasets are provided with three different tiers of access. Users are kindly asked to first agree to the terms of data usage, especially for access to behavioral data, which prohibits identifying individuals on these phenotyping data.

The complete LEMON data can be retrieved from the first location (GWDG) under point 1 (a) below. Moreover, with the complementary project by Mendes *et al*. the raw MRI data are currently available from the OpenNeuro and INDI mentioned under point 1 (b) below. All MRI datasets are shared in NIFTI files, and all anatomical scans have been defaced. A standard ASCII (^∗^.csv, ^∗^.txt) format was employed to upload all other experimental data such as surveys, test batteries, and demographical data in GWDG, and in NITRC.

### 1. Complete MPILMBB LEMON Data

a. The complete LEMON Data can be assessed via Gesellschaft für wissenschaftliche Datenverarbeitung mbH Göttingen (GWDG) https://www.gwdg.de/. Raw and preprocessed data at this location is accessible through web browser https://ftp.gwdg.de/pub/misc/MPI-Leipzig_Mind-Brain-Body-LEMON/ and a fast FTP connection (ftp://ftp.gwdg.de/pub/misc/MPI-Leipzig_Mind-Brain-Body-LEMON/). In the case the location of the data changes in the future, the location of the dataset can be resolved with PID 21.11101/0000-0007-C379-5 (e.g. http://hdl.handle.net/21.11101/0000-0007-C379-5).

b. Additionally, the complete LEMON Data is accessible via Functional Connectomes Project International Neuroimaging Data-Sharing Initiative (INDI) at Child Mind Institute (Data Citation 1).

### 2. Only MPILMBB LEMON MRI Raw Data

The OpenNeuro.org platform also hosts the raw data (Data Citation 2). The OpenNeuro repository provides API access available via https://openneuro.org/dataset/api/. In addition, similar to all other datasets in OpenNeuro, our dataset is available via Amazon Web Services S3 object data store (Data Citation 2).

### 3. Only MPILMBB Behavioral Data

Additionally, the MPILMBB LEMON behavioral data can be found at Neuroimaging Tools and resources Collaboratory (NITRC): https://www.nitrc.org/projects/mpilmbb.

## Additional information

**How to cite this article**: Babayan, A. **et al**. A mind-brain-body dataset of MRI, EEG, cognition, emotion, and peripheral physiology in young and old adults. *Sci. Data*. 6:180308 https://doi.org/10.1038/sdata.2018.308 (2019).

**Publisher’s note**: Springer Nature remains neutral with regard to jurisdictional claims in published maps and institutional affiliations.

## Supplementary Material



Supplementary Information

## Figures and Tables

**Table 1:** Exclusion criteria.

Exclusion criteria via telephone screening: Self-reported
► Diagnosis of hypertension without intake of antihypertensive medication
► Any other cardiovascular disease (current and/or previous heart attack or congenital heart defect)
► History of psychiatric diseases that required inpatient treatment for longer than 2 weeks, within the last 10 years (psychosis, attempted suicide, post-traumatic stress disorder)
► History of neurological disorders (multiple sclerosis, stroke, epilepsy, brain tumor, meningoencephalitis, severe concussion)
► History of malignant diseases
► Intake of one of the following medications (centrally active medication, beta- and alpha-blocker, cortisol, any chemotherapeutic or psychopharmacological medication)
► Positive drug anamnesis (extensive alcohol, MDMA, amphetamines, cocaine, opiates, benzodiazepine, cannabis)
► MRI exclusion criteria (metallic implants, braces, non-removable piercings, tattoos, pregnancy, claustrophobia, tinnitus, surgical operation in the last 3 months)
► Previous participation in any scientific study within the last 10 years
► Previous or current enrollment in undergraduate, graduate or postgraduate psychology studies

**Table 2:** Overview of Measures and Data Availability for Day 0 and Day 1.

Days	Assessments	Details	n
Participant Enrollment Day 0	Telephone Screening/study and MRI/ Medical briefing	Inclusion	n=227
		Exclusion	n=468
Assessment Day 1	Cognitive Test Battery	CVLT	n=228
		TAP	n=227
		TMT	n=227
		WST	n=227
		LPS-2 (Subtest 3)	n=227
		RWT	n=227
	Mood Questionnaire	MDBF (Day 1)	n=227
	Blood Pressure 1	BP1 (left)	n=225
		Pulse1 (left)	n=223
		BP1 (right)	n=223
		Pulse1 (right)	n=222
	Peripheral physiological recordings during rs-fMRI	ECG	n=213
		Blood pressure (beat-to-beat)	n=143
		Pulse (Photoplethysmography)	n=143
		Respiration	n=162
	MRI	Fieldmap for rs-fMRI	n=226
		Spin echo images with reversed phase encoding direction (2 pairs) for rs-fMRI	n=226
		rs-fMRI	n=226
		MP2RAGE	n=226
		T2-weighted	n=224
		FLAIR (2D)	n=110
		FLAIR (3D)	n=115
		DWI (in first 112 participants)	n=110
		Spin echo images with reversed phase encoding direction (2 pairs) for DWI (in first 112 participants)	n=110
		DWI (after 112 participants)	n=115
		Spin echo images with reversed phase encoding direction (2 pairs) for DWI (after 112 participants)	n=115
		SWI and QSM	n=111
	Mind Wandering Questionnaire	NYC-Q	n=227
	Blood Pressure 2	BP2 (left)	n=225
		Pulse2 (left)	n=225
	Peripheral Blood Sample Laboratory Analysis	Electrolytes: Sodium (NA+), Potassium (K), Chloride (Cl-)	n=217
		Liver: Alanine transaminase (ALAT), asparate transaminase (ASAT), Gamma-glutamyltransferase (GGT)	n=217
		Kidney: Creatinine	n=217
		Lipid metabolism: Cholesterol, High density lipoprotein cholesterol (HDL), Low density lipoprotein cholesterol (LDL), triglycerides	n=217
		Inflammatory mediators: C-reactive protein (CRP)	n=217
		Thyroid gland: Thyreotropin/ Thyroid-stimulating hormone (TSH)	n=217
		Glucose (not fasting), Glycated hemoglobin (HbA1c; NSGP/DCCT), Glycated hemoglobin (HbA1c; IFCC)	n=215
		Complete Blood cell Count (CBC) without differential	n=213
		Prothrombin time (PT) & International normalized ratio (INR)	n=211
	Frozen Blood Sample	Serum (Frozen Samples)	n=220
		EDTA full blood (Frozen Aliquots)	n=217
		Blood Sample in RNA tubes (Frozen Samples)	n=211
	Anthropometry	Body weight	n=227
		Body height	n=227
		Waist and hip circumference	n=227
	Hair Sample	Hair 4 cm	n=177
BP1 = Blood Pressure measured before scan, BP2 = Blood Pressure measured after scan, CVLT = California Verbal Learning Task, DWI = Diffusion-weighted imaging, ECG = Electrocardiography, EDTA full blood = Ethylenediaminetetraacetic acid in full blood, FLAIR (2D) = Fluid-attenuated inversion recovery (2 Dimensional), FLAIR (3D) = Fluid-attenuated inversion recovery (3 Dimensional), LPS-2 (Subtest 3) = Subtest 3 of the “Leistungsprüfsystem 2”, MDBF = Multidimensional Mood State Questionnaire, MP2RAGE = Magnetization-Prepared 2 Rapid Acquisition Gradient Echoes, NYC-Q = New York Cognition Questionnaire, RNA = Ribonucleic acid, rsfMRI = Resting-state Magnetic Resonance Imaging, RWT = Regensburger Wortflüssigkeitstest, SWI = T2*/susceptibility-weighted imaging, QSM = quantitative susceptibility mapping, T2-weighted = Spin-Spin-Relaxation imaging, TAP = Test of Attentional Performance, TMT = Trail Making Test, WST = Wortschatztest.			

**Table 3:** Overview of Measures and Data Availability for Day 2.

Days	Assessments	Details	n
Assessment Day 2	Mood Questionnaire	MDBF (Day 2)	n=218
	EEG	Raw rs-EEG (Brain Products ActiCaps)	n=217
		Preprocessed rs-EEG	n=202
		Digitized EEG channel locations	n=144
	Drug Test	Multi 8/2 Drogentest	n=220
	Emotion and Personality Battery	ERQ	n=220
		CERQ	n=220
		PSQ	n=220
		TICS	n=220
		COPE	n=219
		LOT-R	n=220
		STAXI	n=220
		NEO-FFI	n=220
		STAI-G-X2	n=220
		FEV	n=220
		YFAS	n=169
		BIS/BAS	n=220
		UPPS	n=220
		TAS-26	n=220
		MARS	n=208
		F-SozU K-22	n=220
		MSPSS	n=220
		TEIQue-SF	n=220
	Psychiatric screening	SCID I	n=219
		HAM-D	n=218
		AUDIT	n=219
		BSL-23	n=169
AUDIT=Alcohol Use Disorder Identification Test, BIS/BAS=Behavioral Inhibition and Approach System, BSL-23=Borderline Symptoms List (short version), CERQ=Cognitive Emotion Regulation Questionnaire, COPE=Coping Orientations to Problems Experienced, CVLT=California Verbal Learning Task, DWI=Diffusion-weighted imaging, ECG=Electrocardiography, EDTA full blood=Ethylenediaminetetraacetic acid in full blood, ERQ=Emotion Regulation Questionnaire, F-SoZU K-22=Social Support Questionnaire, FEV=Eating Behavior, FLAIR (2D)=Fluid-attenuated inversion recovery (2 Dimensional), FLAIR (3D)=Fluid-attenuated inversion recovery (3 Dimensional), FTP=Future Time Perspective Questionnaire, HAM-D=Hamilton Depression scale (HAM-D), LOT-R=Optimism Pessimism Questionnaire-Revised, MARS=Affect Regulation Style, MDBF=Multidimensional Mood State Questionnaire, MP2RAGE=Magnetization-Prepared 2 Rapid Acquisition Gradient Echoes, MSPSS=Multidimensional Scale of Perceived Social Support, NEO-FFI=Big-Five of Personality, PSQ=Perceived Stress Questionnaire, RNA=Ribonucleic acid, RsEEG=Resting-state electroencephalogram, STAI-G-X2=State-Trait Anxiety Inventory (short version), STAXI=State-Trait Anger Expression Inventory, TAS-26=Toronto-Alexithymia Scale, TEIQue-SF=Emotional Intelligence Questionnaire, TICS=Trier Inventory of Chronic Stress, UPPS=Impulsivity Questionnaires, YFAS=Addicted Eating Behavior.			

**Table 4:** Follow-up assessment days.

Days	Assessments	Details		n
**Follow-up LEMON**	Blood Pressure 3	BP3 (left)		n=159
	Future Time Perspective Questionnaire	FTP		n=141
	Mood Questionnaire	MBDF (Day 3)		n=159
**Additional data from complimentary project by Mendes *et al*.**	Blood Pressure	BP1 (left)		n=91
		Pulse1 (left)		n=44
	Peripheral physiological recordings during rs-fMRI of participants in LEMON and Mendes *et al*. (test-retest sample for LEMON)	ECG	rs-fMRI 1 (AP-run1)	n=98
			rs-fMRI 2 (PA-run1)	n=97
			rs-fMRI 3 (AP-run2)	n=94
			rs-fMRI 4 (PA-run2)	n=90
		Blood pressure (beat-to-beat)	rs-fMRI 1 (AP-run1)	n=101
			rs-fMRI 2 (PA-run1)	n=101
			rs-fMRI 3 (AP-run2)	n=97
			rs-fMRI 4 (PA-run2)	n=91
		Pulse (Photoplethysmography)	rs-fMRI 1 (AP-run1)	n=101
			rs-fMRI 2 (PA-run1)	n=101
			rs-fMRI 3 (AP-run2)	n=97
			rs-fMRI 4 (PA-run2)	n=91
		Respiration	rs-fMRI 1 (AP-run1)	n=98
			rs-fMRI 2 (PA-run1)	n=97
			rs-fMRI 3 (AP-run2)	n=94
			rs-fMRI 4 (PA-run2)	n=90
	Additional peripheral physiological recordings during rs-fMRI of participants in Mendes *et al*.	ECG	rs-fMRI 1 (AP-run1)	n=86
			rs-fMRI 2 (PA-run1)	n=84
			rs-fMRI 3 (AP-run2)	n=80
			rs-fMRI 4 (PA-run2)	n=74
		Blood pressure (beat-to-beat)	rs-fMRI 1 (AP-run1)	n=79
			rs-fMRI 2 (PA-run1)	n=76
			rs-fMRI 3 (AP-run2)	n=74
			rs-fMRI 4 (PA-run2)	n=67
		Pulse (Photoplethys-mography)	rs-fMRI 1 (AP-run1)	n=79
			rs-fMRI 2 (PA-run1)	n=76
			rs-fMRI 3 (AP-run2)	n=74
			rs-fMRI 4 (PA-run2)	n=67
		Respiration	rs-fMRI 1 (AP-run1)	n=86
			rs-fMRI 2 (PA-run1)	n=84
			rs-fMRI 3 (AP-run2)	n=80
			rs-fMRI 4 (PA-run2)	n=74
AP=anterior-posterior phase encoding acquisition (rs-fMRI), BP3 = Blood Pressure measured on follow-up assessment day, FTP = Future Time Perspective Questionnaire, MDBF = Multidimensional Mood State Questionnaire, PA=posterior- anterior hase encoding acquisition (rs-fMRI).				

**Table 5:** Cognitive test battery.

Name	Full Name	Reference	Measured construct
CVLT-part 1	California Verbal Learning Task	Niemann *et al*.^22^	Verbal learning, memory
TAP Alertness	Test of Attentional Performance: Alertness	Zimmermann & Fimm^23^	Alertness, general wakefulness
TAP Incompatibility	Test of Attentional Performance: Incompatibility	Zimmermann & Fimm^23^	Interference
TAP Working Memory	Test of Attentional Performance: Memory	Zimmermann & Fimm^23^	Working memory
TMT A	Trail Making Test	Reitan^24^	Visuomotor speed
TMT B	Trail Making Test	Reitan^24^	Executive function
CVLT-part 2	California Verbal Learning Task	Niemann *et al*.^22^	Verbal learning, memory
WST	Wortschatztest	Schmidt & Metzler^25^	Verbal intelligence, crystallized intelligence
LPS-2, subtest 3	Subtest 3 of the “Leistungsprüfsystem 2”	Kreuzpointner *et al*.^26^	Fluid intelligence
RWT S-Words	Regensburger Wortflüssigkeitstest: S-words	Aschenbrenner *et al*.^27^	Verbal fluency
RWT Animals	Regensburger Wortflüssigkeitstest: Animals	Aschenbrenner *et al*.^27^	Verbal fluency
These tests are completed in a fixed order on assessment day 1.			

**Table 6:** Emotion and personality test battery with their reliability measures.

	Abbr.	Full Name	Reference	Items	Measured construct	M (±SD)	Cronbach's alpha	
							Original	LEMON
1	**NEO-FFI**^**a**^	Neo Five Factor Inventory	Costa & McCrae^30^; Borkenau & Ostendorf^29^	60	Neuroticism	1.5 (±0.6)	0.87	0.81
					Extraversion	2.4 (±0.5)	0.81	0.79
					Openness for Experiences	2.7 (±0.5)	0.75	0.71
					Agreeableness	2.8 (±0.4)	0.72	0.7
					Conscientiousness	2.7 (±0.6)	0.84	0.85
2	**UPPS**	Impulsive Behavior Scale	Whiteside & Lynam^32^; Schmidt *et al*.^31^	45	Lack of Premeditation	22.6 (±3.9)	0.85	0.69
					Urgency	25.8 (±4.8)	0.84	0.8
					Sensation Seeking	32.4 (±7.0)	0.83	0.85
					Lack of Perseverance	19.1 (±4.7)	0.85	0.83
3	**BIS/BAS**	Bahavioural Inhibition and Approach System	Carver & White^34^; Strobel *et al*.^33^	24	Behavioral Inhibition Score (BIS)	19.9 (±3.1)	0.78	0.68
					BAS Subscale Drive	12.0 (±2.1)	0.69	0.74
					BAS Subscale Reward Responsiveness	16.9 (±2.0)	0.69	0.54
					BAS Subscale Fun Seeking	12.3 (±1.7)	0.67	0.47
4	**ERQ**^**a**^	Emotion Regulation Questionnaire	Gross & John^36^; Abler & Kessler^35^	**10**	Reappraisal	4.7 (±1.0)	0.74	0.79
					Suppression	3.7 (±1.2)	0.77	0.71
5	**CERQ**	Cognitive Emotion Regulation Questionnaire	Garnefski *et al*.^38^; Loch *et al*.^37^	27	Acceptance	6.9 (±2.8)	0.6	0.84
					Positive Refocusing	5.0 (±2.7)	0.86	0.82
					Refocusing on Planning	8.0 (±2.6)	0.77	0.78
					Positive Reappraisal	6.8 (±2.8)	0.78	0.74
					Putting into Perspective	7.2 (±2.7)	0.77	0.75
					Self Blame	4.2 (±2.3)	0.73	0.7
					Rumination	5.4 (±2.7)	0.66	0.69
					Catastrophizing	2.6 (±2.3)	0.73	0.73
					Blaming Others	2.3 (±1.8)	0.76	0.76
6	**MARS**^**a**^	Meausre of Affect Regulation Style	Larsen & Prizmic^39^	**38**	Behavioral Distraction	3.1 (±0.8)	0.75	0.69
					Cognitive Distraction	3.5 (±0.9)	0.78	0.67
					Situation-focused Strategies	3.4 (±0.9)	0.6	0.63
					Affect-focused Strategies	2.5 (±0.9)	0.59	0.65
					Disengagement	1.8 (±0.9)	0.55	0.58
					Avoidance	2.5 (±0.8)	0.51	0.57
7	**F-SozU K-22**^**a**^	Social Support Questionnaire	Fydrich *et al*.^41^; Fydrich *et al*.^40^	22	Emotional Support	4.6 (±0.5)	0.81-0.93	0.88
					Practical Support	4.5 (±0.6)	0.81-0.93	0.79
					Social Integration	4.2 (±0.6)	0.81-0.93	0.71
					Availability of Trusted Person	4.7 (±0.6)	0.81-0.93	0.72
					Satisfaction with Social Support	3.6 (±0.9)	0.81-0.93	0.58
8	**MSPSS**	Multidimensional Scale of Perceived Social Support	Zimet, G.D *et al*.^42^	12	Friends	24.3 (±3.8)	0.85	0.93
					Family	22.7 (±5.3)	0.87	0.92
					Significant Others	25.2 (±3.6)	0.91	0.87
9	**COPE**	Coping Orientations to Problems Experienced (Brief COPE)	carver^44^; Knoll *et al*.^43^	**28**	Self Distraction	5.2 (±1.4)	0.71	0.67
					Active Coping	5.7 (±1.3)	0.68	0.66
					Denial	2.1 (±1.2)	0.54	0.61
					Substance Use	2.7 (±1.2)	0.9	0.91
					Use of Emotional Support	5.9 (±1.5)	0.71	0.78
					Use of Instrumental Support	5.5 (±1.7)	0.64	0.91
					Behavioral Disengagement	3.0 (±1.0)	0.65	0.21
					Venting	4.3 (±1.3)	0.5	0.57
					Positive Reframing	5.5 (±1.5)	0.64	0.73
					Planning	6.2 (±1.2)	0.73	0.44
					Humor	4.1 (±1.6)	0.73	0.68
					Acceptance	5.5 (±1.5)	0.57	0.68
					Religion	2.8 (±1.4)	0.82	0.69
					Self-Blame	4.6 (±1.5)	0.69	0.75
10	**LOT-R**	Life Orientation Test – Revised	Scheier *et al*.^46^;Glaesmer *et al*.^45^	10	Optimism	8.9 (±2.3)	0.69	0.75
					Pessimism	4.7 (±2.2)	0.68	0.66
					Overall Score	27.3 (±4.8)	0.59	0.62
11	**PSQ**^**a**^	Perceived Stress Questionnaire	Levenstein *et al*.^48^; Fliege *et al*.^47^	20	Worries	26.3 (±18.6)	0.86	0.82
					Tension	28.2 (±19.0)	0.84	0.83
					Joy	67.0 (±18.1)	0.85	0.74
					Demands	32.9 (±20.4)	0.8	0.83
12	**TICS**	Trier Inventory of Chronic Stress	Schulz & Schlotz^50^; Schulz *et al*.^49^	57	Work Overload	9.5 (±6.7)	0.9	0.92
					Social Overload	7.1 (±4.5)	0.84	0.81
					Pressure to Perform	14.0 (±6.4)	0.9	0.87
					Work Discontent	10.6 (±5.7)	0.85	0.86
					Excessive Demands at Work	5.7 (±4.3)	0.87	0.89
					Lack of Social Recognition	4.2 (±3.1)	0.84	0.84
					Social Tension	5.4 (±3.7)	0.87	0.86
					Social Isolation	6.6 (±4.6)	0.88	0.86
					Chronic Worrying	5.3 (±3.6)	0.88	0.88
13	**FEV**	Three-factor Eating Questionnaire	Stunkard & Messick^51^; Pudel & Westenhöfer^52^	60	Cognitive Restraint	6.5 (±4.7)	0.87	0.86
					Disinhibition	4.8 (±2.6)	0.82	0.66
					Hunger	4.6 (±2.9)	0.78	0.71
14	**YFAS**	Yale Food Addiction Scale	Gearhardt *et al*.^54^; Meule *et al*.^53^	27	YFAS Symptom Count^b^	1.4 (±0.7)	0.86	0.31^c^
15	**TEIQue-SF**^**a**^	Emotional Intelligence Questionnaire	Petrides & Furnham^56^; Freudenthaler *et al*.^57^	30	Well-being	5.6 (±0.9)	0.94	0.79
					Self Control	5.1 (±0.8)	0.86	0.52
					Emotionality	5.0 (±0.8)	0.9	0.61
					Sociability	4.9 (±0.8)	0.88	0.56
					Global Trait Emotiona Intelligence	152.4 (±18.0)	0.96	0.86
16	**STAI-G-X2**	State-Trait Anxiety Inventory	Spielberger *et al*.^59^; Laux *et al*.^58^	20	Trait Anxiety	36.3 (±8.6)	0.88-0.94	0.91
17	**TAS**	Toronto Alexithymia Scale	Bagby *et al*.^63^; Kupfer *et al*.^62^	26	Difficulty with Identifying Feelings	13.2 (±4.2)	0.84	0.79
					Difficulty with Expressing and Describing Feelings	13.7 (±3.9)	0.69	0.76
					Externally-oriented Thinking	14.4 (±3.4)	0.67	0.63
18	**STAXI**	State-Trait Anger Expression Inventory	Spielberger^61^; Schwenkmezger *et al*.^60^	44	Trait Anger	18.3 (±4.1)	0.88	0.79
					Anger-In	16.4 (±3.9)	0.79	0.76
					Anger-Out	11.7 (±3.2)	0.86	0.81
					Anger-Control	22.8 (±3.8)	0.88	0.76
19	**MDBF**	Multidimensional Mood State Questionnaire	Steyer *et al*.^64^	24	Good-Bad	34.8 (±3.0)	0.91-0.94	0.82-0.87
					Awake-Tired	31.6 (±4.6)	0.92-0.94	0.92-0.93
					Calm-Nervous	32.8 (±3.6)	0.86-0.91	0.84-0.87
20	**FTP**^**a**^	Future Time Perspective Questionnaire	Lang and Carstensen^65^	10	Future Time Perspective	44.8 (±11.3)	0.92	0.73
21	**NYC-Q**	New York Cognition Questionnaire	Gorgolewski *et al*.^66^	31	NYC-Q Score	NA	NA	NA
^a^Scores on these 7 questionnaires have been calculated as mean scores (NEO- FFI, ERQ, MARS, F-SozU K-22, PSQ standardized means, TEIQue-SF subscales, FTP). All the rest are calculated as sum scores.								
^b^Symptom counts indicate how many of 7 YFAS-criteria were fulfilled (range 0–7).								
^c^Internal consistency based on Kuder-Richardson’s alpha coefficient (Meule & Gearhardt,^112^).								
Note: These first 18 questionnaires are completed in randomized order on a computer (LimeSurvey) on assessment day 2. The last three questionnaires (19–21) are completed as paper and pen version on a follow-up assessment day.								

**Table 7:** MRI sequences.

MRI scan	Sequence parameters	File name (raw, nifti format)	File name (preprocessed, nifti format)
Gradient echo fieldmap for rs-fMRI distortion correction	voxel size=2.3 mm isotropic, FOV=202 mm, imaging matrix=88×88, 64 slices with 2.3 mm thickness, TR= 680 ms, TE1=5.19 ms, TE2=7.65 ms, flip angle=60°, bandwidth=389 Hz/pixel, prescan normalization, no partial fourier, duration=2 min 3 s	acq-GEfmap_run-01_magnitude1, acq-GEfmap_run-01_magnitude2, acq-GEfmap_run-01_phasediff	
Spin echo EPI with reversed phase encoding for rs-fMRI distortion correction	voxel size=2.3 mm isotropic, FOV=202 mm, imaging matrix=88×88, 64 slices with 2.3 mm thickness, TR=2200 ms, TE=50 ms, flip angle=90°, echo spacing=0.67ms, phase encoding=A ≫ P / P ≫ A, bandwidth=1776 Hz/pixel, partial fourier 6/8, no pre-scan normalization, duration=29 s each	acq-SEfmapBOLDpost_dir-AP_epi, acq-SEfmapBOLDpost_dir-PA_epi, acq-SEfmapBOLDpre_dir-AP_epi, acq-SEfmapBOLDpre_dir-PA_epi	
Resting-state fMRI (T2*-weighted gradient-echo EPI BOLD)	Axial acquisition orientation, phase encoding=A ≫ P, voxel size=2.3 mm isotropic, FOV=202 mm, imaging matrix=88×88, 64 slices with 2.3 mm thickness, TR=1400 ms, TE=30 ms, flip angle=69°, echo spacing=0.67 ms, bandwidth=1776 Hz/pixel, partial fourier 7/8, no pre-scan normalization, multiband acceleration factor=4, 657 volumes, slice order=interleaved, duration=15 min 30 s	acq-AP-run-01_bold	task-rest_acq-AP_run-01_native, task-rest_acq-AP_run-01_MNI2mm
Magnetization Prepared 2 Rapid Acquisition Gradient Echoes (MP2RAGE)	Sagittal acquisition orientation, one 3D volume with 176 slices, TR=5000 ms, TE=2.92 ms, TI1=700 ms, TI2=2500 ms, FA1=4°, FA2=5°, pre-scan normalization, echo spacing=6.9 ms, bandwidth=240 Hz/pixel, FOV=256 mm, voxel size= 1 mm isotropic, GRAPPA acceleration factor 3, slice order=interleaved, duration=8 min 22 s	acq-mp2rage_T1w, acq-mp2rage_T1map, acq-mp2rage_defacemask, inv-1_mp2rage,inv-2_mp2rage	acq-mp2rage_brain
T2-weighted	Sagittal acquisition orientation, one 3D volume with 176 slices, TR=3200 ms, TE=409 ms, FA=variable, pre-scan normalization, echo spacing=3.42 ms, bandwidth=751 Hz/pixel, FOV=256 mm, voxel size=1 mm isotropic, GRAPPA acceleration factor 2, duration=4 min 43 s	T2w	
Fluid-attenuated inversion recovery (FLAIR) 2D (scanned in first 112 participants)	Axial acquisition orientation, 28 slices, TR=10000 ms, TE=90 ms, TI=2500 ms, FA=180°, pre-scan normalization, echo spacing=9.98 ms, bandwidth=199 Hz/pixel, FOV=220 mm, voxel size=0.9×0.9×4.0 mm^3^, slice order=interleaved, duration=4 min 42 s	acq-lowres_FLAIR	
3D SPACE sequence with fluid-attenuated inversion-recovery preparation (introduced after first 112 participants)	Sagittal acquisition orientation, one 3D volume with 192 slices, TR=5000 ms, TE=395 ms, TI=1800 ms, FA=variable, pre-scan normalization, echo spacing=3.36 ms, bandwidth=781 Hz/pixel, FOV=250 mm, voxel size=1 mm isotropic, GRAPPA acceleration factor 2, duration=7 min 2 s	acq-highres_FLAIR	
Diffusion-weighted Imaging (DWI, scanned in first 112 participants)	88 axial slices, voxel size=1.7 mm isotropic, 60 diffusion-encoding gradient directions, b-value of 1000 s/mm^2^, 7 non-diffusion-weighted b0 distributed in the sequence, TR=7000 ms, TE=80 ms, FA=90°, bandwidth=1502 Hz/pixel, echo spacing=0.78 ms, FOV=220 mm, voxel dimension=1.7 mm isotropic, imaging matrix=128×128, acquired with ⅞ partial Fourier encoding and GRAPPA (acceleration factor 2, 32 ref. lines), 60 diffusion-encoding gradient directions, b-value=1000 s/mm^2^, 7 b0 images, raw data filter, fat suppression (strong), advanced shim mode, no prescan normalization, interleaved acquisition, CMRR sequence, monopolar diffusion scheme, SENSE coil combine, multiband acceleration factor 2, phase encoding A ≫ P, duration=9 min 27 s	dwi	
Spin echo images with reversed phase encoding for DWI distortion correction (scanned in first 112 participants)	Two volumes with A ≫ P and P ≫ A phase encoding, voxel size=1.7 mm isotropic, 88 axial slices, TR=7000 ms, TE=80 ms, FA=90°, bandwidth=1502 Hz/pixel, echo spacing 0.78 ms, FOV=220 mm, voxel dimension=1.7 mm isotropic, imaging matrix=128×128, acquired with ⅞ partial Fourier encoding and GRAPPA (acceleration factor 2, 32 ref. lines), fat suppression (strong), advanced shim mode, no prescan normalization, interleaved acquisition, CMRR sequence, SENSE coil combine, multiband acceleration factor 2, duration=1 min 59 s each	acq-SEfmapDWI_dir-AP_epi, acq-SEfmapDWI_dir-PA_epi	
Diffusion-weighted Imaging (DWI, new version introduced after first 112 participants)	88 axial slices, voxel size=1.7 mm isotropic, 60 diffusion-encoding gradient directions, b-value of 1000 s/mm^2^, 7 non-diffusion-weighted b0 distributed in the sequence, TR=7000 ms, TE=80 ms, FA=90°, bandwidth=1502 Hz/pixel, echo spacing=0.78 ms, FOV=220 mm, voxel dimension=1.7 mm isotropic, imaging matrix=128×128, acquired with ⅞ partial Fourier encoding and GRAPPA (acceleration factor 2, 32 ref. lines), 60 diffusion-encoding gradient directions, b-value=1000 s/mm^2^, 7 b0 images, raw data filter, fat suppression, advanced shim mode, no prescan normalization, interleaved acquisition, CMRR sequence, monopolar diffusion scheme, SENSE coil combine, multiband acceleration factor 2, phase encoding A ≫ P, duration=8 min 38 s	dwi	
Spin echo images with reversed phase encoding for DWI distortion correction (new version introduced after first 112 participants)	Two volumes with A ≫ P and P ≫ A phase encoding, voxel size=1.7 mm isotropic, 88 axial slices, TR=7000 ms, TE=80 ms, FA=90°, bandwidth=1502 Hz/pixel, echo spacing 0.78 ms, FOV=220 mm, voxel dimension=1.7 mm isotropic, imaging matrix=128×128, acquired with ⅞ partial Fourier encoding and GRAPPA (acceleration factor 2, 32 ref. lines), fat suppression, advanced shim mode, no prescan normalization, interleaved acquisition, CMRR sequence, SENSE coil combine, multiband acceleration factor 2, duration=1 min 10 s each	acq-SEfmapDWI_dir-AP_epi, acq-SEfmapDWI_dir-PA_epi	
Gradient echo Susceptibility-weighted data for SWI and QSM estimation (introduced after first 112 participants)	Axial acquisition orientation, one 3D volume with 160 slices, TR=30 ms, TE=17.3 ms, FA=13°, bandwidth=150 Hz/pixel, FOV=205 mm, acquired with 6/8 phase partial Fourier and GRAPPA (acceleration factor 2, 24 ref. lines), no prescan normalization, interleaved acquisition, voxel size=0.8 mm isotropic, duration=7 min 50 s	acq-phase_GRE, acq-mag_GRE	
