# Supplementary material for: A mind-brain-body dataset of MRI, EEG, cognition, emotion, and peripheral physiology in young and old adults
Source: Sci Data. 2019 Feb 12;6:180308. doi: 10.1038/sdata.2018.308 (PMC6371893; doi:10.1038/sdata.2018.308)
Supplement: Supplementary Information [file sdata2018308-s2.docx]

Supplementary Information

**A mind-brain-body dataset of MRI, EEG, cognition, emotion, and peripheral physiology in young and old adults**

Correspondence to: babayan@cbs.mpg.de

**This file includes:**

**Supplementary Figure S1:** Cross-subject coverage of fMRI and DWI (p. 2)

**Supplementary Figure S2:** Density plots of scores on emotion and personality variables for both age groups (younger and older adults) (p. 3)

**Supplementary Table S1:** Table of common participants between our study and Mendes et al. (p. 9)

**Supplementary Figure S1: Cross-subject coverage of fMRI and DWI**


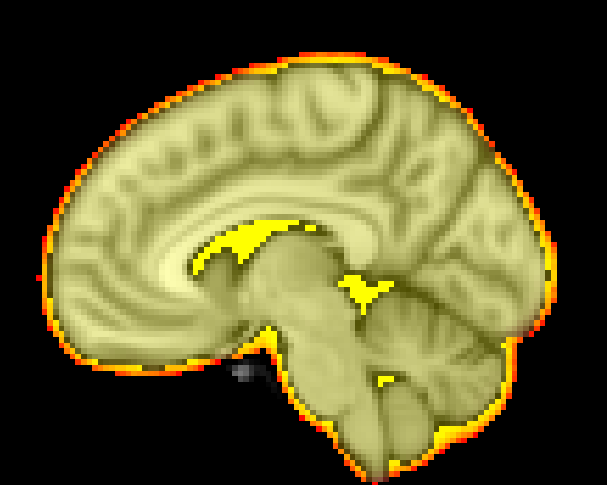

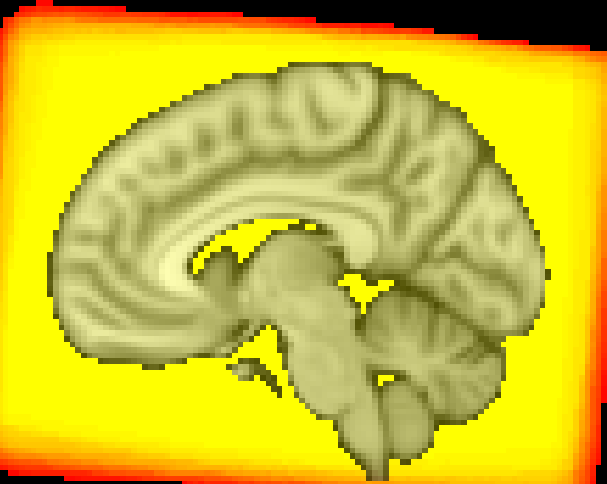


Supplementary Figure S1

The figure shows the cross-subject coverage of the fMRI (left) and DWI (right) data normalized to the MNI brain. The yellow-red color map represents the range of 100% overlap (yellow) to 80% overlap (red). The fMRI data show the coverage after individually masking the brain (provided preprocessed data).

| **Supplementary Figure S2:** **Density plots of scores on emotion and personality variables for both age groups (younger and older adults)**  **NEO-FFI** |
| --- |
| 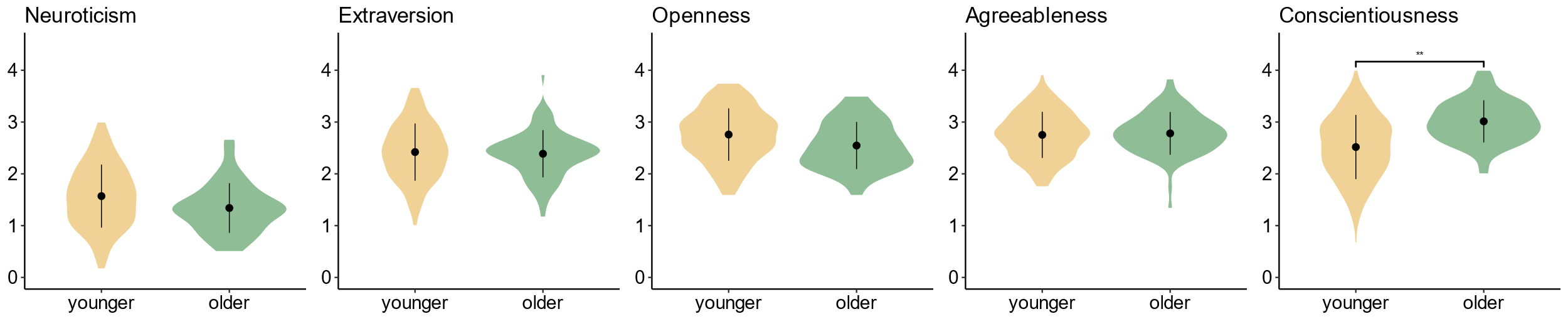 |
| **STAI & STAXI** |
| 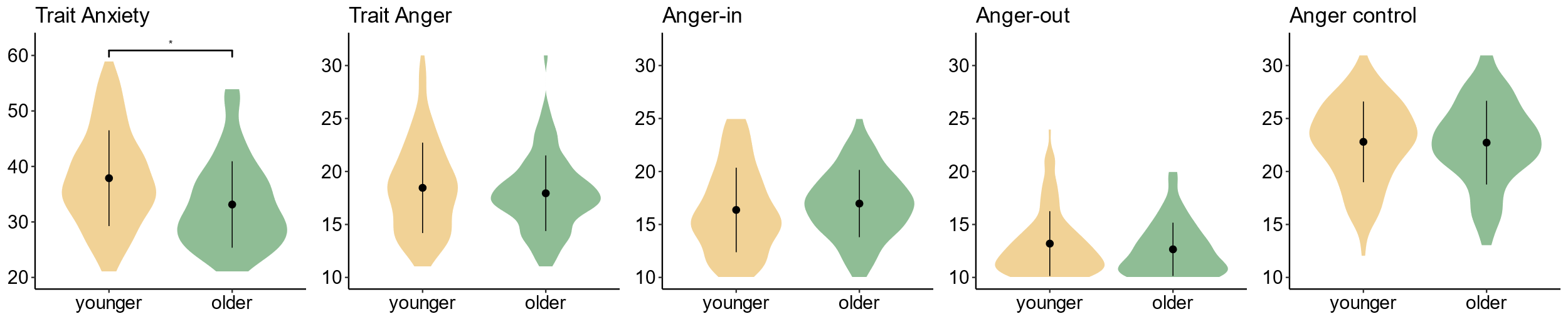 |
| **LOT-R** |
| 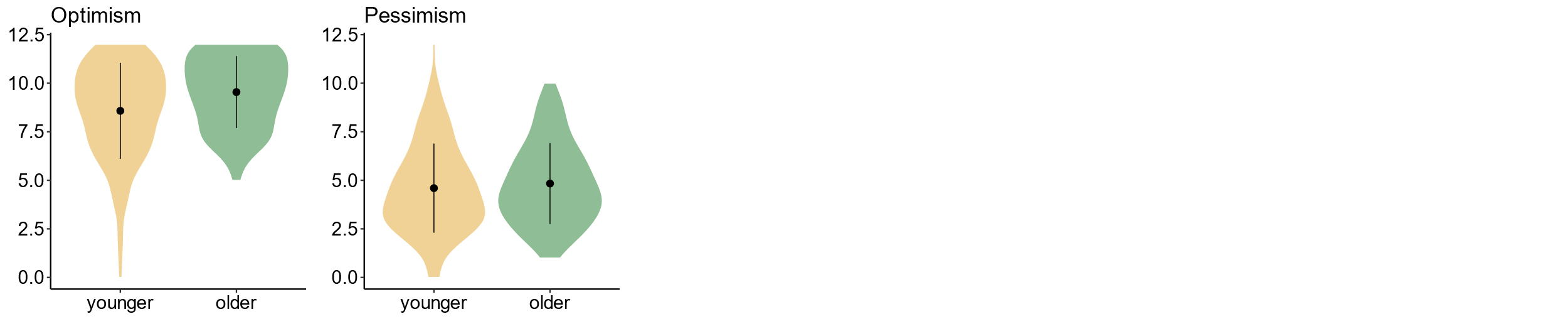 |
| **UPPS** |
| 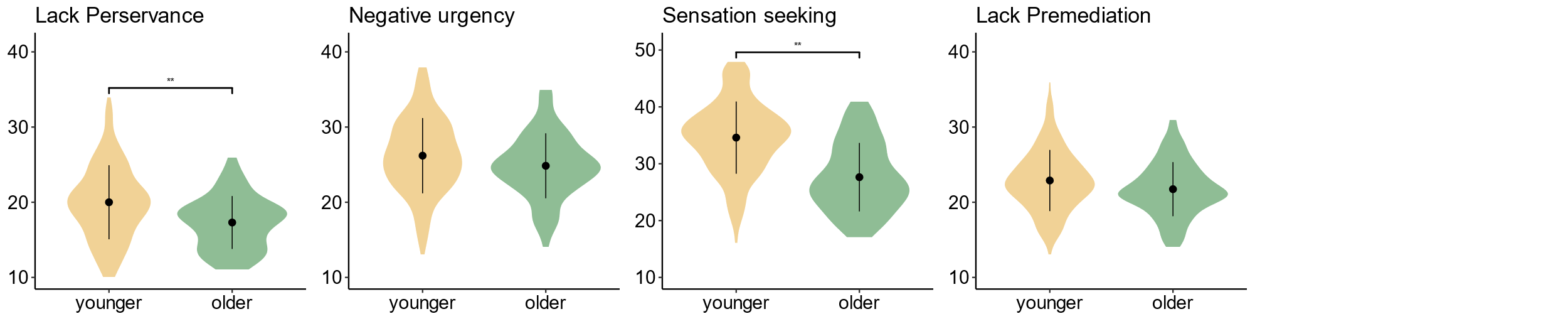 |

| **BIS/BAS** |
| --- |
| 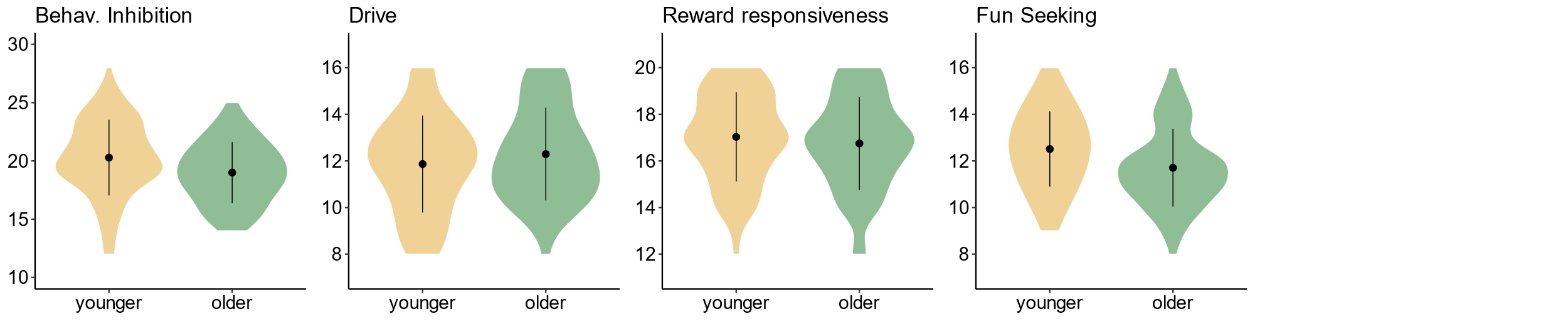 |
| **CERQ** |
| 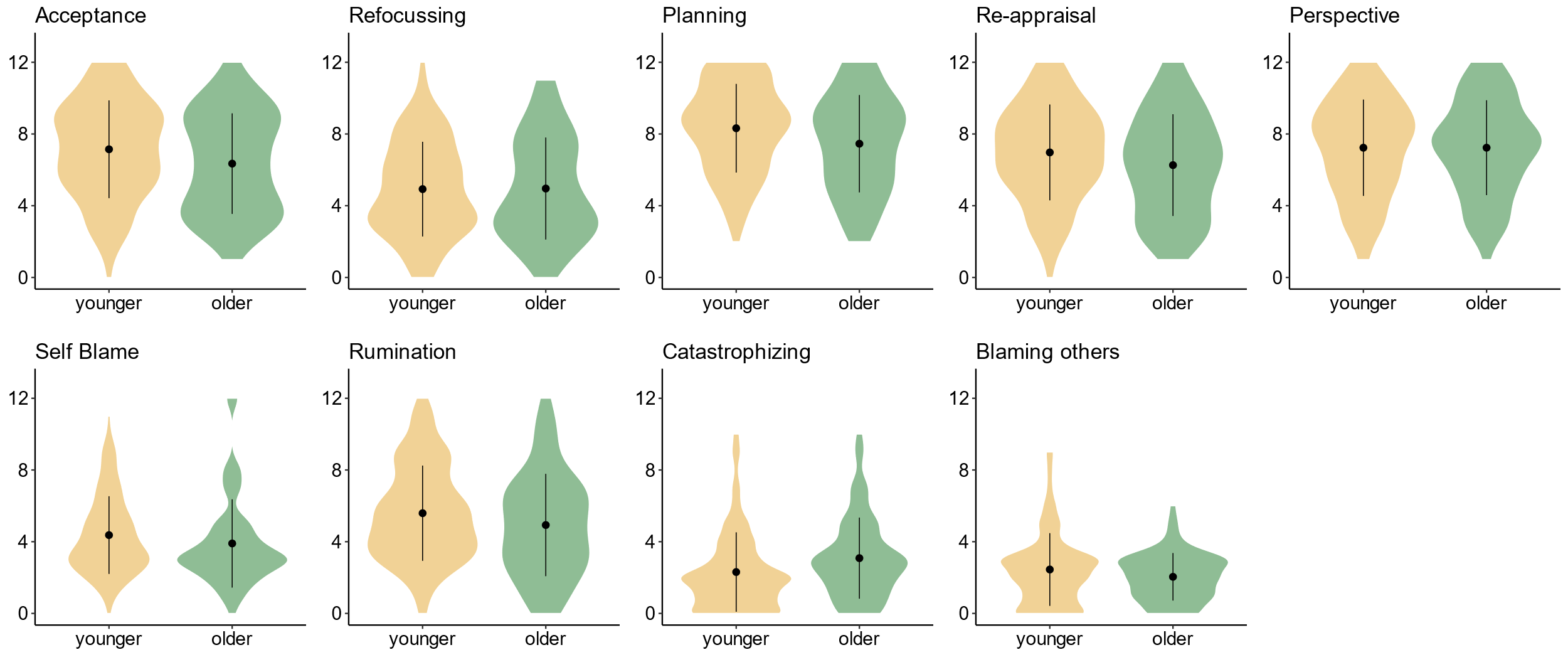 |
| **COPE** |
| 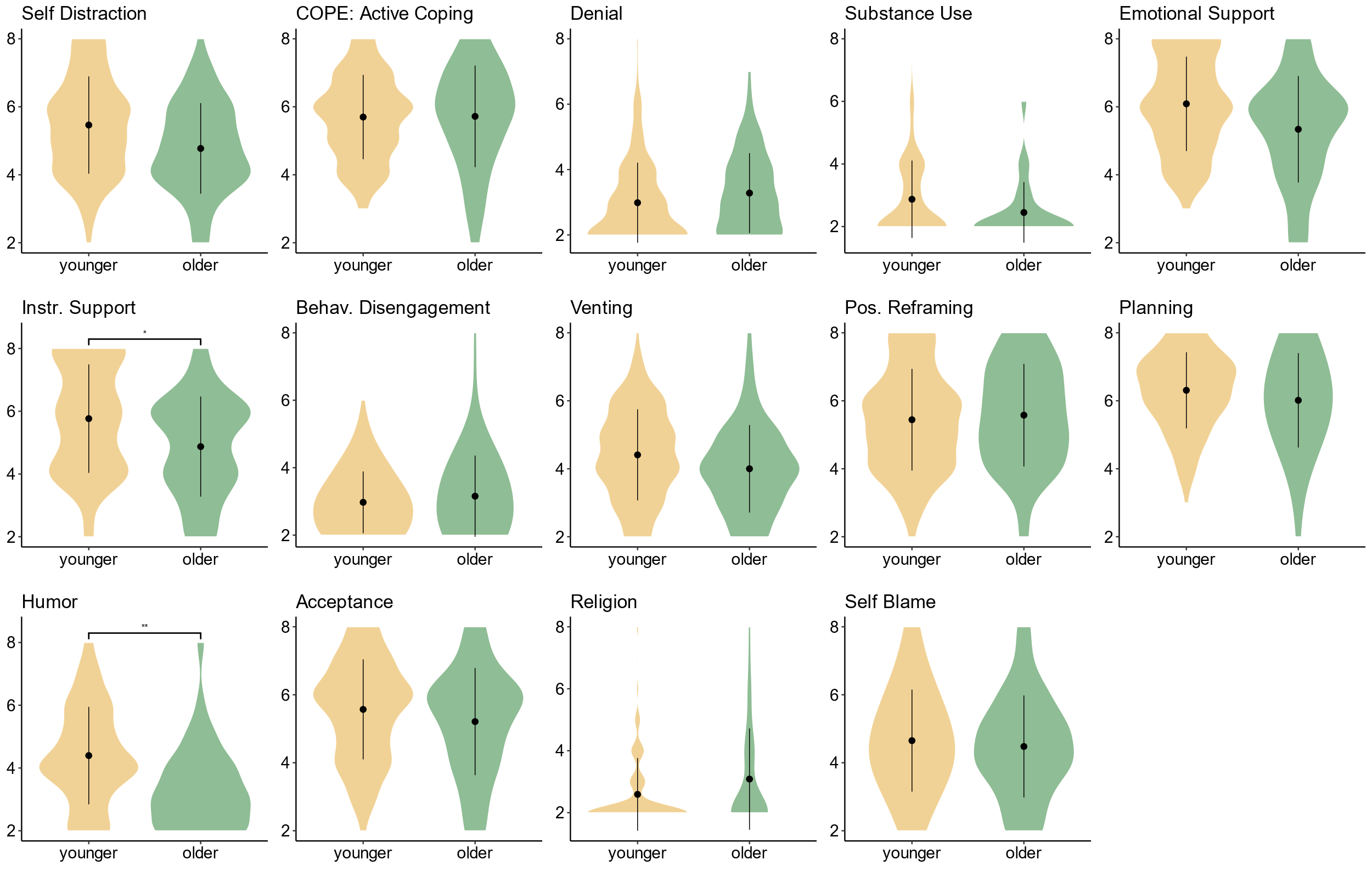 |
| **ERQ** |
| 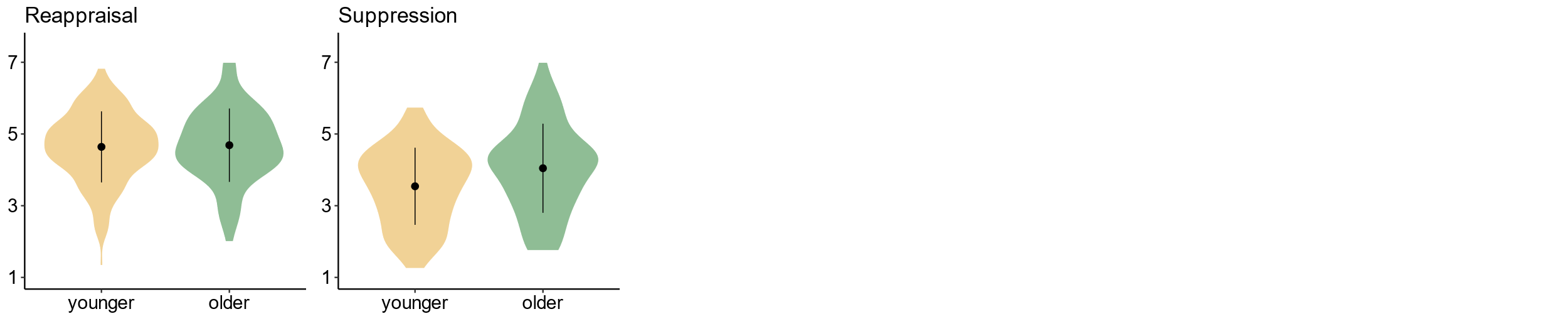 |
| **MARS** |
| 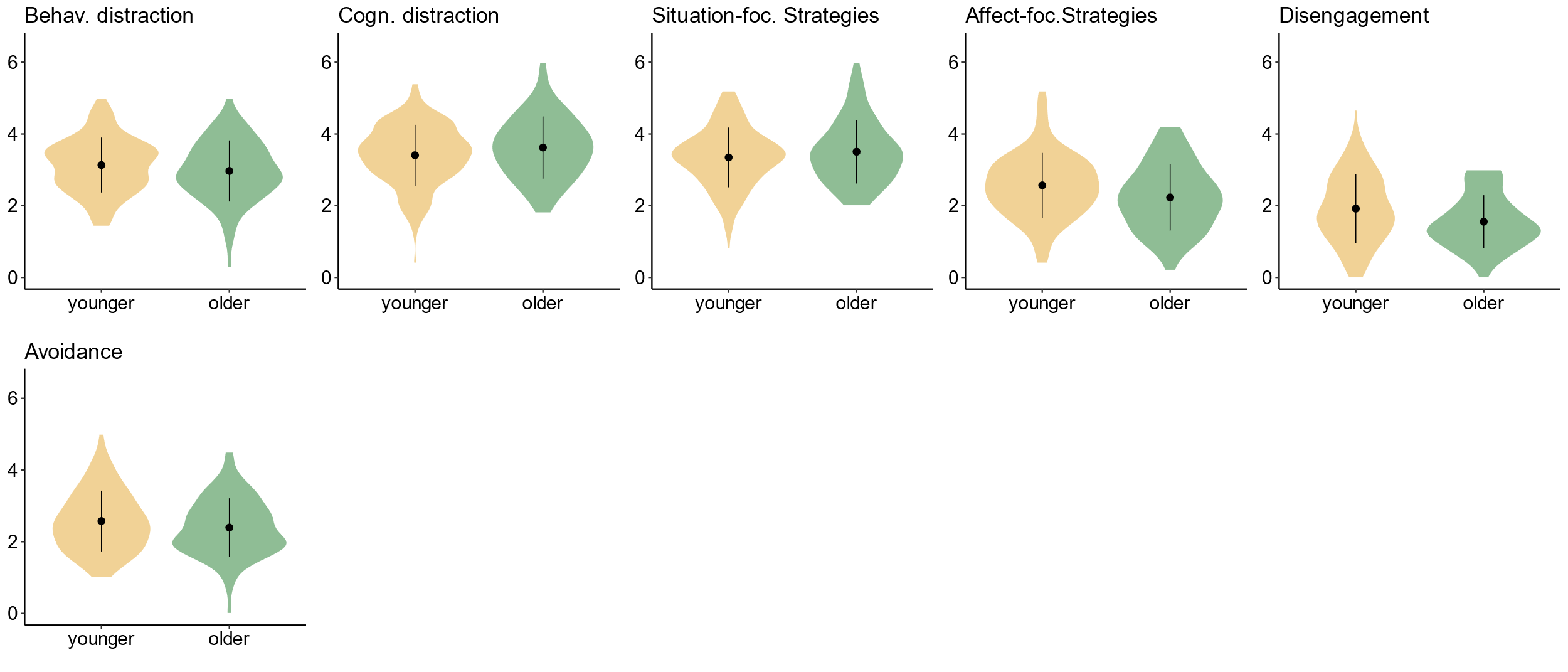 |
| **F-SozU** |
| 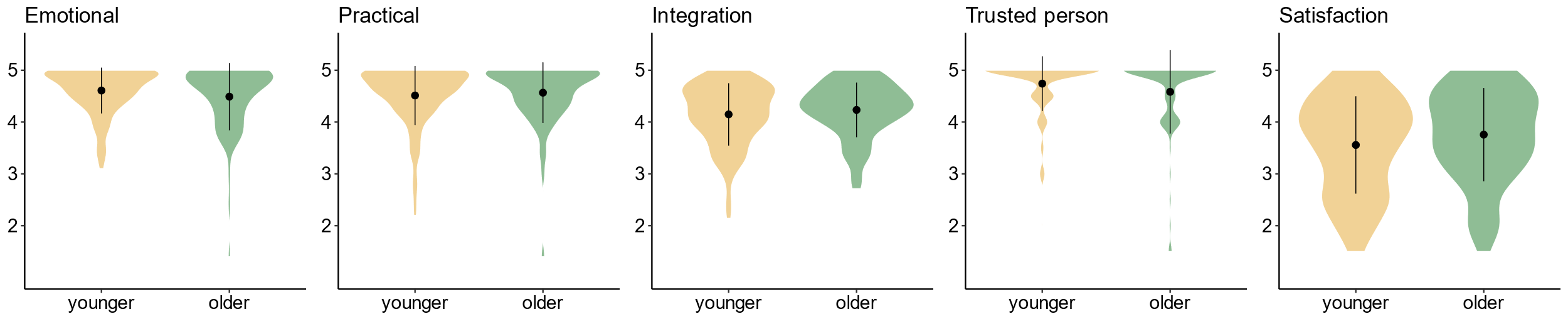 |
| **MSPSS** |
| 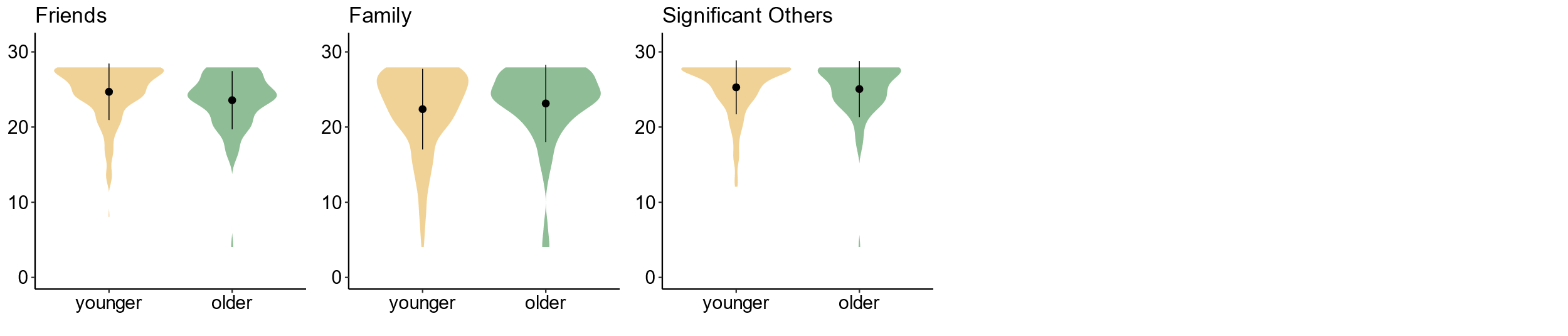 |

| **PSQ** |
| --- |
| 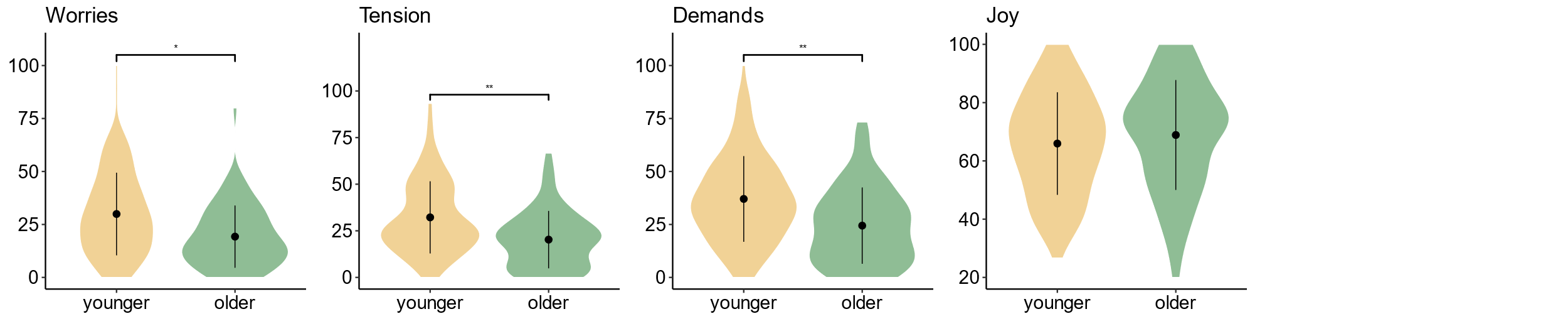 |
| **TICS** |
| 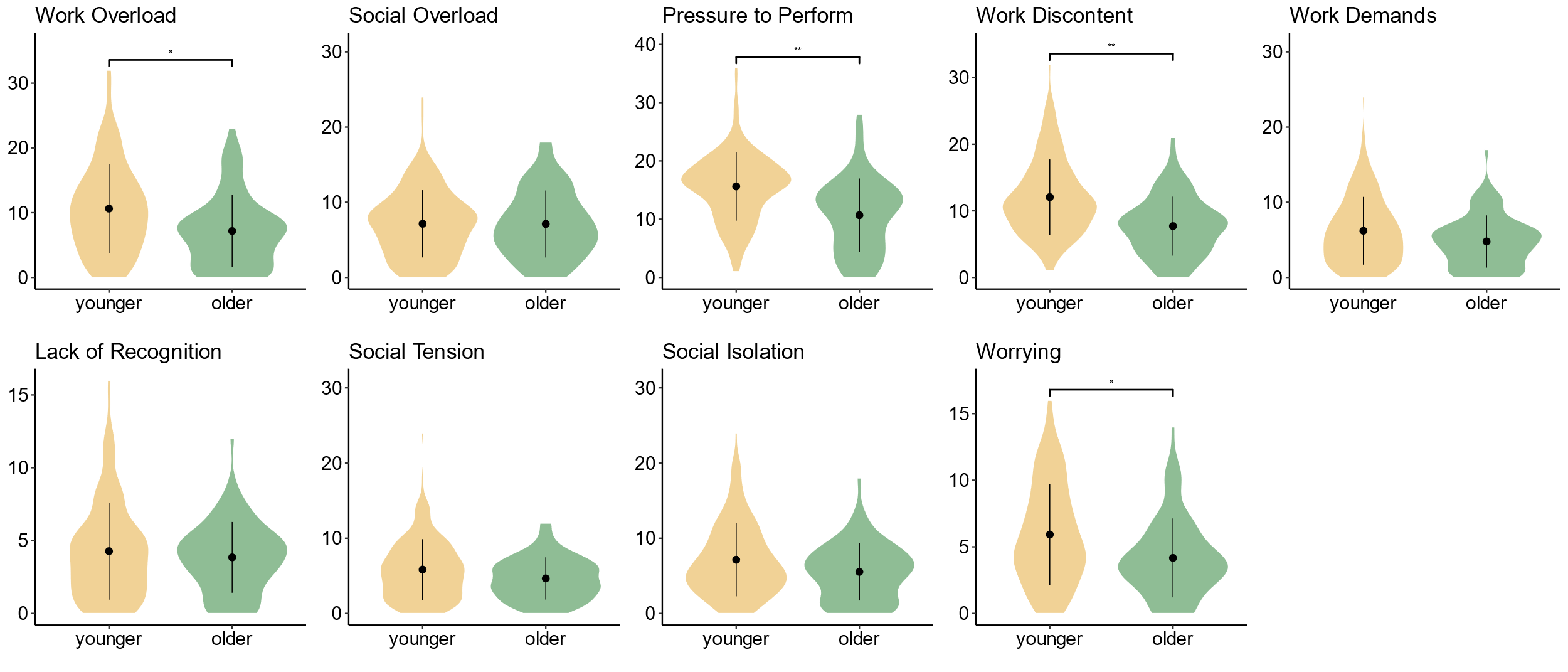 |
| **FEV** |
| 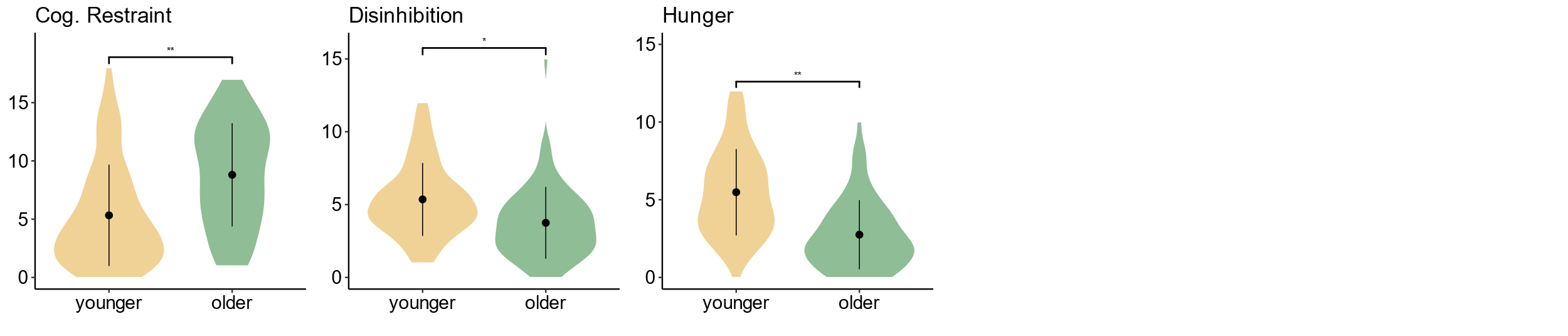 |
| **TEIQuesF** |
| 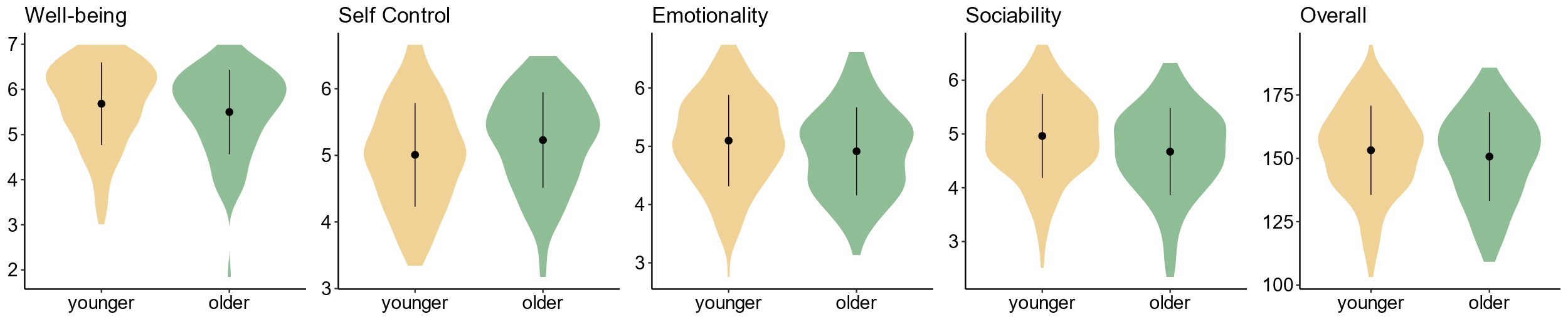 |

| **TAS** |
| --- |
| 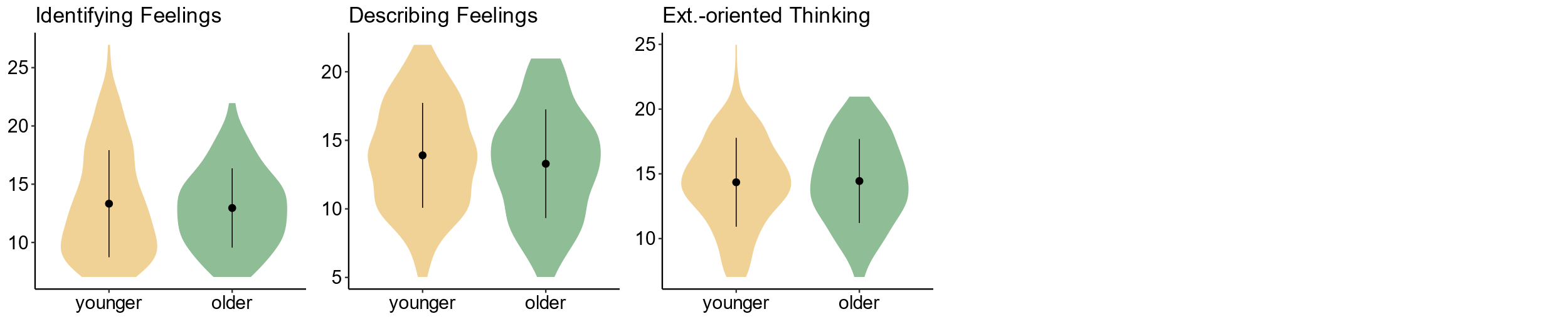 |
| **MDBF Day 1** |
| 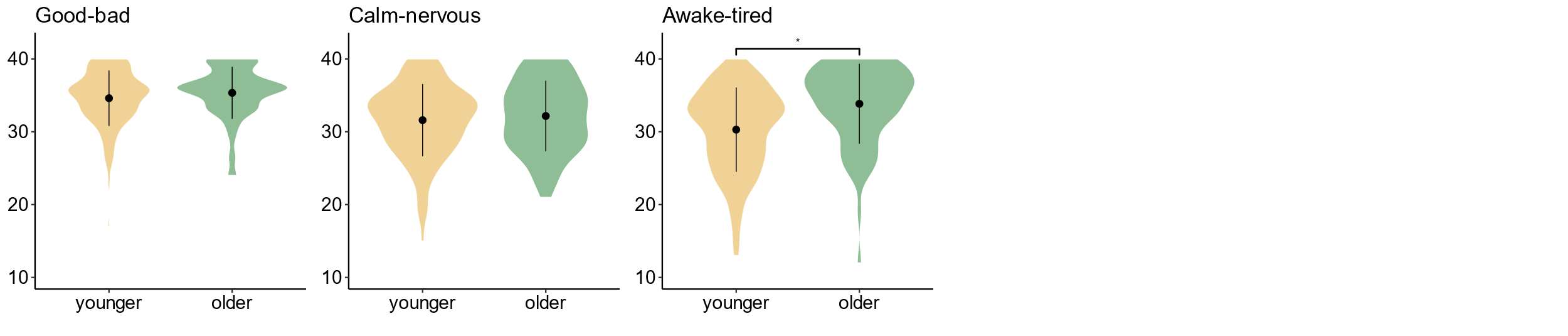 |
| **MDBF Day 2** |
| 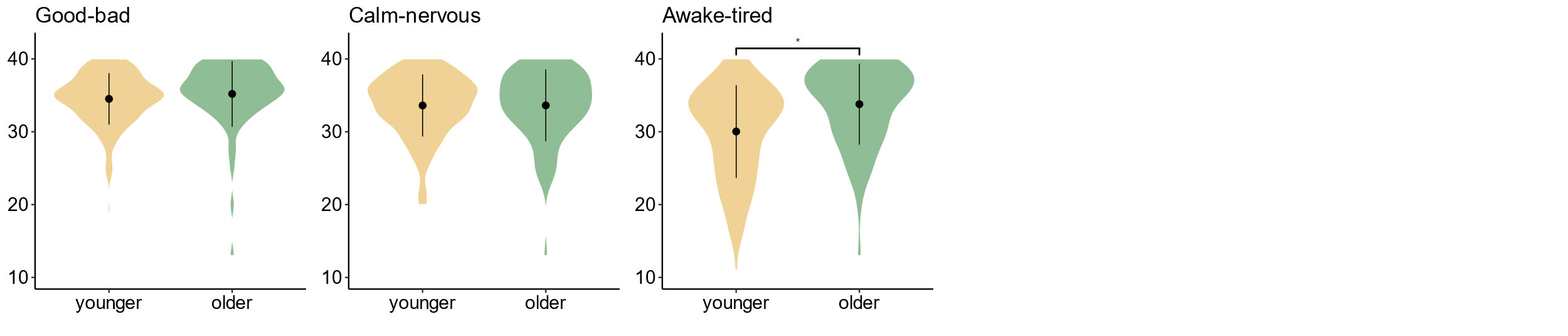 |
| **MDBF Day 3** |
| 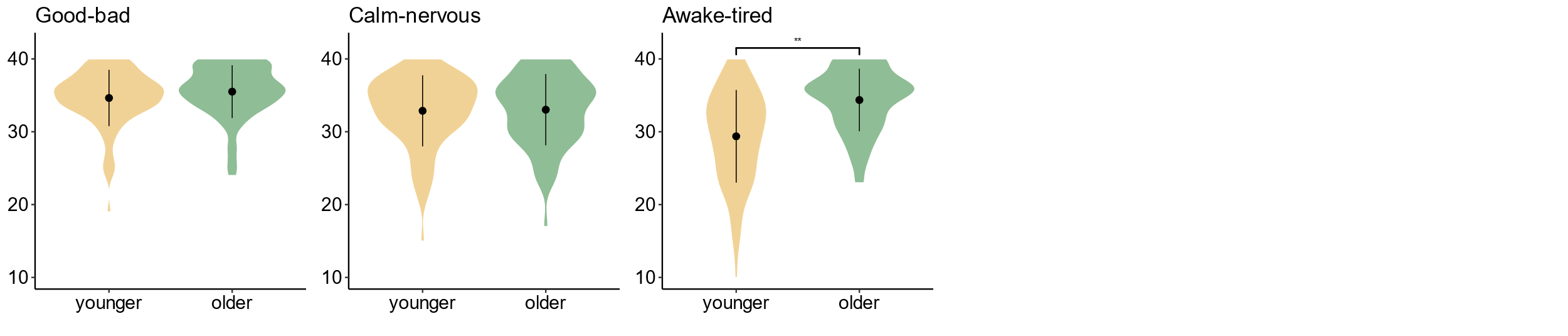 |
| **FTP** |
| 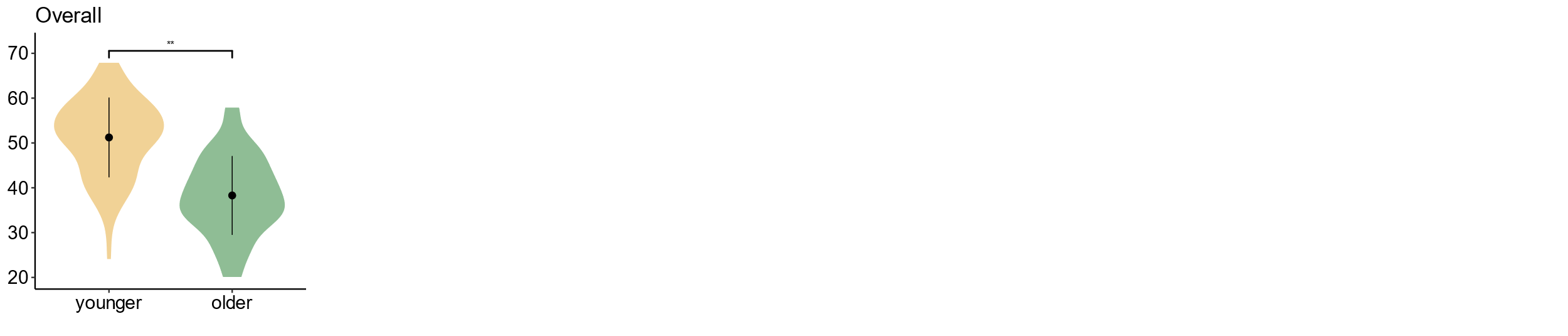 |
| The figure of Supplementary Figure S2 represents violin plots of all emotion battery subscales for younger and older adults. The violins display the distribution of the data (on each side). Points and error bars within the violins represent mean ± SDs. Asterisks indicate significant age-group differences based on unpaired Welch *t*-tests after Bonferroni correction (**p* < 0.0005, ***p* < 0.00001). Please refer to Table 6 within the main paper for questionnaire-related abbreviations. |

**Supplementary Table S1: Table of common participants** **between our study and Mendes et al.**

sub-010002 sub-010035 sub-010065 sub-010087 sub-010164 sub-010224

sub-010004 sub-010036 sub-010067 sub-010088 sub-010165 sub-010225

sub-010005 sub-010037 sub-010068 sub-010090 sub-010166 sub-010226

sub-010006 sub-010038 sub-010069 sub-010091 sub-010168 sub-010227

sub-010007 sub-010040 sub-010070 sub-010092 sub-010191 sub-010228

sub-010015 sub-010041 sub-010071 sub-010093 sub-010192 sub-010229

sub-010016 sub-010042 sub-010072 sub-010094 sub-010194 sub-010231

sub-010017 sub-010044 sub-010073 sub-010126 sub-010195 sub-010232

sub-010020 sub-010045 sub-010074 sub-010136 sub-010196 sub-010233

sub-010022 sub-010046 sub-010075 sub-010137 sub-010200

sub-010023 sub-010048 sub-010076 sub-010138 sub-010201

sub-010024 sub-010050 sub-010077 sub-010141 sub-010203

sub-010026 sub-010051 sub-010078 sub-010142 sub-010204

sub-010027 sub-010052 sub-010079 sub-010146 sub-010210

sub-010029 sub-010056 sub-010080 sub-010148 sub-010213

sub-010030 sub-010059 sub-010081 sub-010152 sub-010214

sub-010031 sub-010060 sub-010083 sub-010155 sub-010215

sub-010032 sub-010061 sub-010084 sub-010157 sub-010216

sub-010033 sub-010062 sub-010085 sub-010162 sub-010218

sub-010034 sub-010064 sub-010086 sub-010163 sub-010220

The table shows the common participants (n=109) between Mendes et al. ^21^ and our study, and who completed the full procedure of both studies and have available data.
